# Comprehensive assessment of precious metal concentration, distribution, and recovery potential in municipal solid waste incineration residues from northern Vietnam

**DOI:** 10.1039/d5ra08421k

**Published:** 2026-01-13

**Authors:** Thi Thu Thuy Nguyen, Truong Xuan Vuong, Thi Thu Ha Pham, Anh Quoc Hoang, Minh Binh Tu, Thi Hue Nguyen

**Affiliations:** a Faculty of Natural Sciences and Technology, TNU-University of Science Tan Thinh Ward Thai Nguyen City 24000 Vietnam xuanvt@tnus.edu.vn; b University of Science, Vietnam National University, Hanoi 19 Le Thanh Tong Hanoi 11000 Vietnam; c Institute of Environmental Technology, Vietnam Academy of Science and Technology 18 Hoang Quoc Viet Street, Cau Giay Hanoi Vietnam

## Abstract

The incineration process of municipal solid waste produces residues which serve as valuable secondary resources for extracting precious metals including Ag, Au, Pt, Pd, and Rh. The research delivers the initial full dataset about metal concentrations and distribution and recovery potential in three residue types which include fine particulate matter (PM_10_), and fly ash (FA), and bottom ash (BA) from incineration facilities in northern Vietnam. The analysis of all samples occurred through ICP-MS to determine the metal content. Scanning electron microscopy coupled with energy-dispersive X-ray spectroscopy (SEM-EDX) was used to characterize particle morphology and elemental associations in the samples. Statistical correlation analysis was applied to identify relationships among metals and to determine how their potential sources differed across municipal, mixed, and industrial waste streams. The metal concentrations showed average values between 1.92 and 10.8 µg Nm^−3^ in PM10, 0.055 and 8.52 mg kg^−1^ in FA, and between 0.097 and 10.7 mg kg^−1^ in BA with Ag showing the highest levels. These concentrations were higher than that of many incineration plants in Japan and Poland, and significantly higher than mean crustal compositions. Among the analysed elements, Ag contributed the largest proportion (17–95%), followed by Au (5–57%), Pd (0–44%), Pt (2–41%), and Rh (<10%). Metal distribution patterns varied with waste composition: Ag was prevalent across all feedstocks, whereas Au was enriched in municipal and mixed waste, and Pt, Pd, and Rh were mainly associated with industrial residues. Strong Pearson correlations (*r* = 0.64–0.94) among Ag/Pd/Pt, Au/Pt, and Pt/Pd/Rh pairs indicate potential co-occurrence and a shared origin. Two economic recovery models demonstrated that both fly ash and bottom ash constitute feasible sources for precious-metal recycling. The results provide a scientific basis for waste valorisation, resource recovery, and circular-economy strategies for MSWI residues in Vietnam.

## Abstract 2

Precious-metal concentrations in PM_10_, fly ash, and bottom ash from MSWI in Northern Vietnam show strong inter-metal correlations and significant recovery potential, supporting waste valorization and circular-economy strategies.

## Graphical abstract

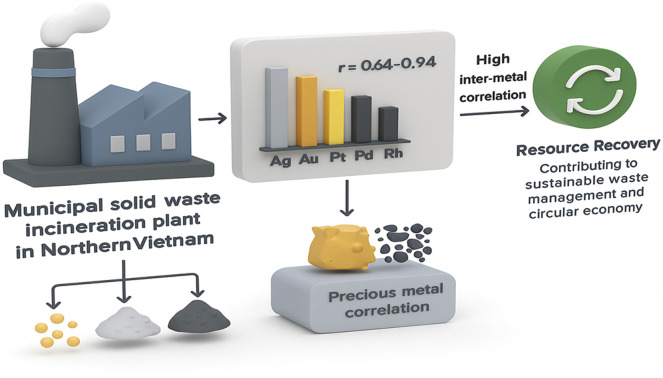

## 1. Introduction

Municipal solid waste (MSW) management has become a worldwide concern because generation of wastes is increasing steadily and is estimated to be around 3.88 billion tonnes by 2050.^1,2^ Waste management is poor in most developing nations, such as Vietnam, because the countries have inadequate finances, obsolete treatment technologies, incomplete regulations, and poor awareness of the community.^3^

In this scenario, incineration of the wastes has been progressively engineered as a very efficient way to reduce the amounts of wastes, remediate the hazardous constituents, and produce heat/electricity. Although municipal solid waste incineration (MSWI) is not inherently environmentally benign, modern incineration systems equipped with advanced air-pollution-control technologies are considered more environmentally sustainable than uncontrolled landfilling or open dumping.^4^ MSWI significantly reduces waste volume (up to 70–90%), destroys hazardous organic compounds, generates usable heat and electricity,^5^ and produces residues from which valuable metals can be recovered.^6,7^ These combined benefits position MSWI as a more controlled and resource-efficient waste-management option within a circular-economy framework.^3,8^ Despite this efficiency, the incineration process itself produces a significant amount of by-products in the form of bottom ash (BA) and fly ash (FA), which together constitute about 20–25% of the original weight of input wastes.^9^

Different research studies have described the chemical composition of MSWI by-products,^10,11^ which commonly contain heavy metals such as Pb, Zn, Cu, Cd, Cr, and Ni, in addition to valuable precious metals including Ag, Au, Pt, Pd, and Rh.^12–14^ Concentrations of these metals in bottom ash are often several times higher than their average crustal abundances, for example, Ag (∼0.07 mg kg^−1^), Au (∼0.004 mg kg^−1^), Pd (∼0.015 mg kg^−1^), Pt (∼0.005 mg kg^−1^), and Rh (∼0.001 mg kg^−1^), and may reach or even exceed levels found in low-grade ores.^15^ Muchova *et al.* (2009) reported approximately 10 mg kg^−1^ Ag and 0.4 mg kg^−1^ Au in bottom ash collected in Amsterdam (the Netherlands), with precious metals occurring across all particle-size fractions below 20 mm.^13^ Similarly, Morf *et al.* (2013) showed that MSWI residues may contain Ag and Au at concentrations suitable for profitable recovery under appropriate treatment schemes.^8^ More recently, Chuchro *et al.* (2025) demonstrated that several European incinerator ashes contain elevated levels of Ag, Au, Pd, and Pt compared with natural geogenic background values,^14^ further confirming the potential of MSWI residues as an emerging secondary metal resource.

Over the past decades, numerous studies have characterized the heavy-metal content of MSWI bottom ash (BA) and fly ash (FA) across different regions. Commonly detected metals include Zn, Pb, Cu, Cr, Ni, Cd, Hg and others, often at high concentrations (*e.g.*, Zn and Pb typically dominate the heavy-metal load).^16^ For example, a recent comprehensive survey of FA reported Zn contents ranging from 2200 to 25 000 mg kg^−1^, Pb between several hundred to over 13 000 mg kg^−1^, and Cu up to 7200 mg kg^−1^ depending on incinerator type and waste composition.^17^ Another recent local study from northern Vietnam confirmed that MSWI ashes, both FA and BA, commonly contain heavy metals such as Pb, Cr, As, Cd, Cu, and Zn, with a large fraction residing in mobile or leachable chemical forms, raising environmental concern.^18^ These findings have led to a broad body of work focusing on heavy-metal risk assessment, environmental leaching, stabilization, and reuse of ash (*e.g.*, as construction material after stabilization or solidification).^19^

While heavy metals have been extensively studied in MSWI ashes, interest has recently grown in the occurrence of precious and technology-critical metals (*e.g.*, Ag, Au, Pt, Pd, Rh), which may accumulate from electronic waste, industrial catalysts, and other waste streams. However, systematic measurements targeting such metals remain relatively rare compared to heavy-metal surveys, especially studies that compare multiple residue types (fly ash, bottom ash, and particulate fines) within a single waste-stream or incinerator system.^1^ Moreover, the growing demand for these precious and critical metals, driven by applications in electronics, catalytic converters, renewable energy, and green technologies, has motivated recent research toward resource recovery from incineration residues, positioning MSWI ash as a potential “urban mine”.^1^

In parallel with measurement and characterization, a variety of treatment and recovery techniques for heavy and valuable metals from MSWI ash have been developed. These include acid leaching (chemical extraction), thermal treatment (incineration/volatilization), stabilization/solidification (cement-based or vitrification), and more advanced methods such as bioleaching or electrokinetics for metal recovery from fly ash.^20^ For instance, recent reviews highlight chemical stabilization and immobilization as widely used approaches to mitigate environmental risks of heavy metals, while thermal separation (with or without chlorination) and acid extraction remain among the most effective routes for metal recovery from fly ash.^17^ However, many of these methods focus on common heavy metals (Pb, Zn, Cu, *etc.*), and their effectiveness for precious-metal recovery is still underexplored, especially for ashes from waste streams with mixed industrial and municipal inputs, such as those typical in Vietnam.

The present study focuses on the precious metals Ag, Au, Pd, Pt, and Rh because they represent economically valuable and technologically critical elements with expanding demand in both current and future industrial applications. Ag and Au remain essential in electronics, photovoltaics, and high-precision manufacturing, while Pd, Pt, and Rh are indispensable components of automotive catalytic converters, hydrogen production technologies, fuel cells, and emerging green-energy processes. Due to their high market value, supply risks, and increasing global consumption, these metals are widely recognized as priority targets for recovery from secondary resources such as MSWI residues.

Even though fly ash from MSWI is generally considered hazardous waste because it often contains dioxins and furans and soluble salts, recent studies have brought into focus its prospect of serving as a source of precious and technology-critical elements (TCEs).^20^ Shen *et al.* (2025) reported that most precious metals in fly ash occur predominantly in the residual fraction, indicating low solubility and limited recoverability using conventional leaching processes.^20^ Likewise, Tian *et al.* (2025) found that fly ash is enriched in base metals such as Pb, Zn, Cd, and Cu, while also containing trace precious metals. This suggests both the need for stabilizing toxic elements and the potential for recovering valuable metals.^21^

The results from the experiments have also ratified the presence of precious metals in incineration ashes.^22–26^ Beikmohammadi *et al.* (2023) directly measured Ag and Au in fly ash at lower levels than in bottom ash but high enough to show the need to consider fly ash in the urban mining scenario for MSWI ashes.^1^ From a multi-element point of view, the work by Fabricius *et al.* (2020) recognized the presence of Ag in the group of the technology-critical elements found in the fly ash and thereby further enhanced the idea that the ashes may have valuable contents besides the traditional heavy metals.^25^

Whereas multiple studies across different regions have reported elevated concentrations of precious metals in MSWI bottom ash and fly ash (*e.g.*, ref. 1, 8, 13 and 14), few investigations have performed a whole-scale statistical comparison across the three main residue streams (bottom ash, fly ash, and particulate fines) to comprehensively evaluate differences in concentration, partitioning, and recovery potential.^13^ No study has yet provided a full-range quantitative comparison of the distribution and recovery potentials for precious metals from Vietnamese MSWI systems, where wastes' composition and operational conditions significantly depart from their equivalents in the West or Japan. This research provides the very first full dataset for the concentrations of five precious metals (Ag, Au, Pt, Pd, and Rh) and their distribution patterns and recovery potentials in the incineration wastes across Vietnamese MSWI plants. The significance of this research lies in addressing a major knowledge gap in the characterization of precious metals in MSWI residues. Previous studies have typically quantified precious metals in either fly ash or bottom ash individually, but only a very limited number have simultaneously investigated their distribution across multiple residue streams such as PM_10_, fly ash, and bottom ash.^1,8^ To date, no comprehensive dataset exists for Vietnamese MSWI plants, despite the fact that their mixed municipal-industrial waste composition differs markedly from that of Europe or Japan and may therefore influence metal content and partitioning.^3^ Conducting a quantitative comparison of metal concentrations, distribution patterns, and recovery potentials across these residue types is essential for identifying feasible precious-metal recovery options and for improving the efficiency of resource-recovery technologies.^9,13^ This study therefore provides the first integrated and comparative dataset for Vietnam, offering an important scientific and practical foundation for evaluating both environmental and economic opportunities for precious-metal recovery within the country's emerging circular-economy framework.

**Fig. 1:**
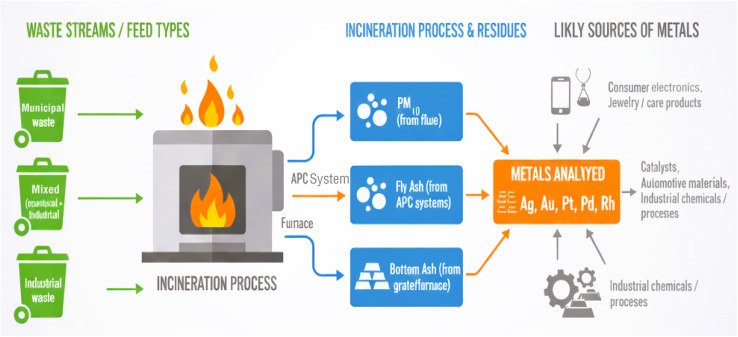
Schematic overview illustrating the relationships between waste-feed types (municipal, mixed, industrial), incineration residues (PM_10_, fly ash, bottom ash), the analyzed precious metals (Ag, Au, Pt, Pd, Rh), and their potential origin sources (electronics, personal care products, catalysts, automotive materials, and industrial chemicals).

Complementing this background, the overall object of the present research work is a thorough evaluation of the occurrence prevalence, patterns of distribution, and recovery potential of precious metals in incineration ashes of Vietnamese municipally generated wastes. For achieving this end, the research work entailed the conduct of the following three major research activities: (i) the measurement of the levels of some chosen precious metals (Ag, Au, Pt, Pd, and Rh) in fine particulate mater (PM), fly ash (FA), and bottom ash (BA); (ii) the characteristics of the distribution and the between-metal relationships of the by-product streams; and (iii) assessing the environmental and economic sustainability of recovery of these metals from MSWI ashes. To improve clarity, a schematic overview summarizing the connections between waste-feed types, incineration residues, investigated metals, and their potential sources has been added (Fig. 1).

## 2. Materials and methods

### 2.1. Materials

**Fig. 2:**
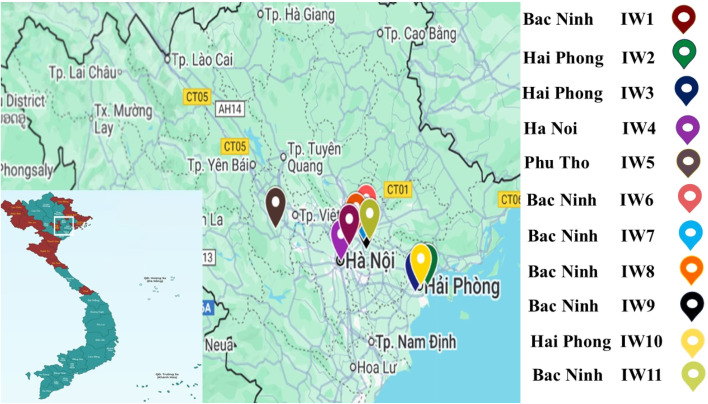
Location of the samples collected from waste incineration plants located across northern Vietnam.

A total of 32 samples were collected from waste incineration plants located across northern Vietnam, comprising fine particulate matter (PM_10_, collected from flue gas), fly ash (FA), and bottom ash (BA). The geographical locations of the sampled facilities are shown in Fig. 2. Among them, five plants treat industrial waste, four treat municipal solid waste (MSW), and two process mixed waste streams (approximately 30% MSW and 70% industrial waste). Sampling was conducted between June and September 2023, with detailed information on each facility provided in Table S1 in SI. The MSWI plant selected for this study is located in northern Vietnam and represents one of the region's major waste-to-energy facilities. It processes mixed municipal and industrial waste streams typical of urban areas in Vietnam, making it regionally representative in terms of waste composition, incineration conditions, and the characteristics of the resulting ash residues. Therefore, the measured concentrations and distribution patterns of precious and heavy metals in PM_10_, fly ash, and bottom ash are directly relevant to the regional context and reflect conditions typical of Vietnamese MSWI operations.

Bottom ash samples were collected directly from the ash outlets at the bottom of the furnace, and fly ash samples were collected from the air pollution control systems. Sampling was carried out following the Vietnamese National Standard for ash sampling (MOST, 2017).^27^ Fine particulate samples (PM_10_) were collected from the incinerator flue gas using an isokinetic sampling system (C5000 ESC, USA) in accordance with the U.S. Environmental Protection Agency Method 201A (EPA, 2020).^28^ The system consisted of a coaxial dust sampling probe equipped with a filter holder, a vacuum pump, a control console, and a gas flow meter. The sampling flow rate was maintained at 30 L min^−1^. The flue gas velocity was determined based on the difference between total and static pressures, and the nozzle diameter of the coaxial probe was adjusted to match the gas flow velocity. Downstream of the probe, cylindrical silica-fiber filters (Whatman GF-25, 90 mm diameter) were used to collect PM_10_ samples efficiently.

### 2.2. Methods

#### 2.2.1. Digestion of fine particle and fly ash samples

**Fig. 3:**
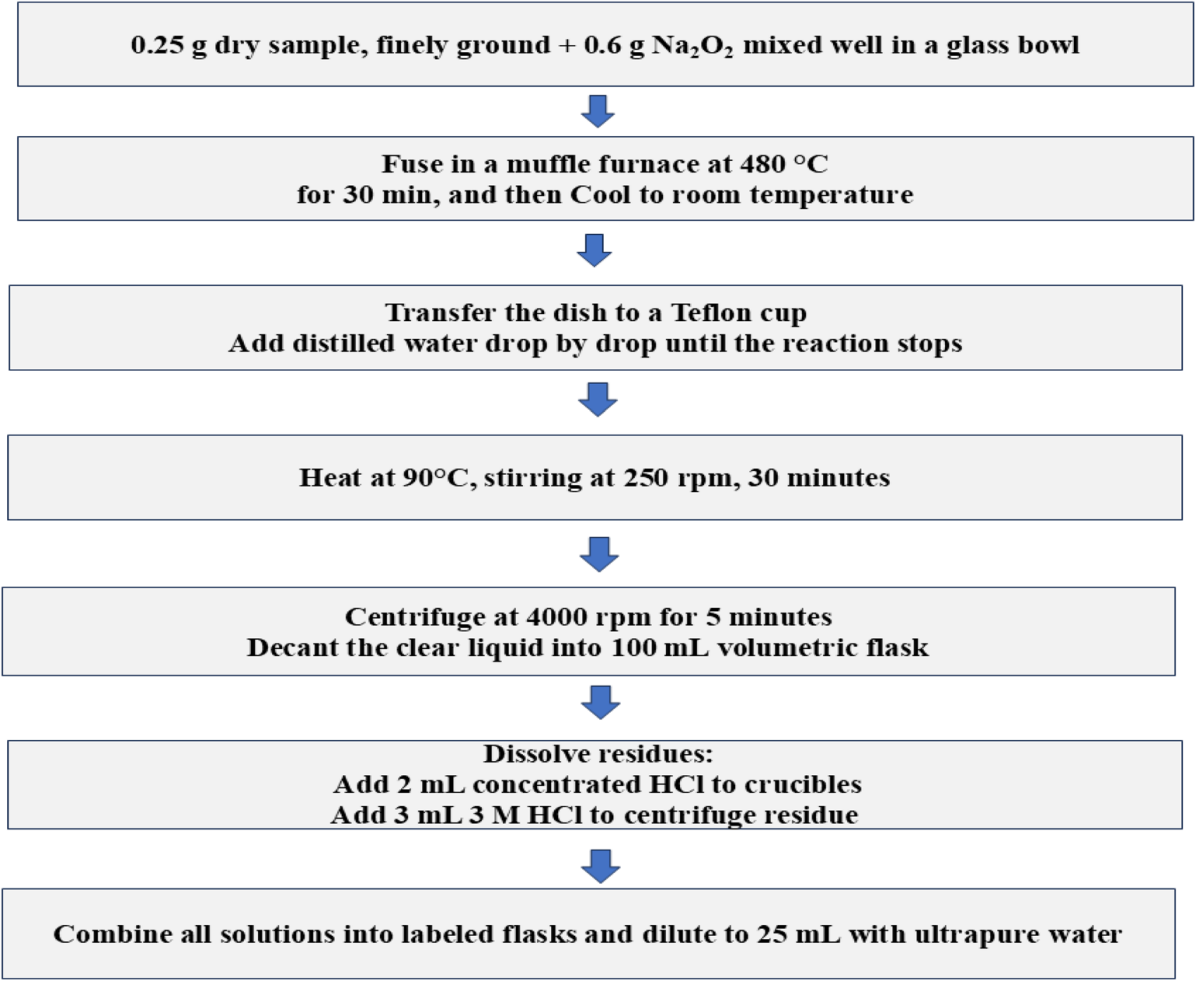
Schematic overview of the digestion process of PM_10_ and fly-ash samples.

The samples were oven-dried at 105 °C for 24 hours prior to homogenization and further analysis. Approximately 0.25 g of oven-dried, finely ground sample was weighed into a nickel or zirconium crucible and thoroughly mixed with about 0.6 g of sodium peroxide (Na_2_O_2_) using a glass stirring rod. Sodium-peroxide fusion was selected because MSWI-derived PM_10_ and fly ash typically contain refractory mineral phases (silicates, aluminates, spinels and metal oxides) that resist complete dissolution by conventional acid digestion. The crucible was then placed in a muffle furnace and heated at 480 °C for 30 min to fuse the sample. During heating at 480 °C for 30 min, Na_2_O_2_ acts as a strong oxidizing and alkaline flux, breaking down the mineral lattice and converting silicate and aluminate matrices into water-soluble sodium silicates (Na_2_SiO_3_), sodium aluminates (NaAlO_2_), and oxidized metal salts, thereby facilitating near-complete recovery of both major and trace elements, including PGEs and Au. This approach is widely used and validated for geological, slag and ash matrices prior to ICP-MS/ICP-OES analysis.^29,30^ After cooling, the fused material was transferred to a Teflon beaker, and an appropriate acid solution was carefully added to dissolve the fused mass. The beakers were subsequently heated at 90 °C on a magnetic stirrer at 250 rpm for 30 min to ensure complete dissolution. Approximately 0.25 g of oven-dried, finely ground sample was weighed into glass crucibles and thoroughly mixed with about 0.6 g of sodium peroxide (Na_2_O_2_) using a glass stirring rod. The resulting suspensions were centrifuged at 4000 rpm for 5 min. The clear supernatant was decanted into 100 mL volumetric flasks. To dissolve any remaining precipitates, 2 mL of concentrated HCl and 3 mL of 3 mol per L HCl were successively added to the Teflon beakers and the residues from the centrifugation step, respectively. All solutions were then combined, transferred into labeled flasks, and diluted to 25 mL with ultrapure water.^28^ A schematic overview of the digestion procedure for PM_10_ and fly-ash samples is presented in Fig. 3.

#### 2.2.2. Digestion of bottom ash samples

**Fig. 4:**
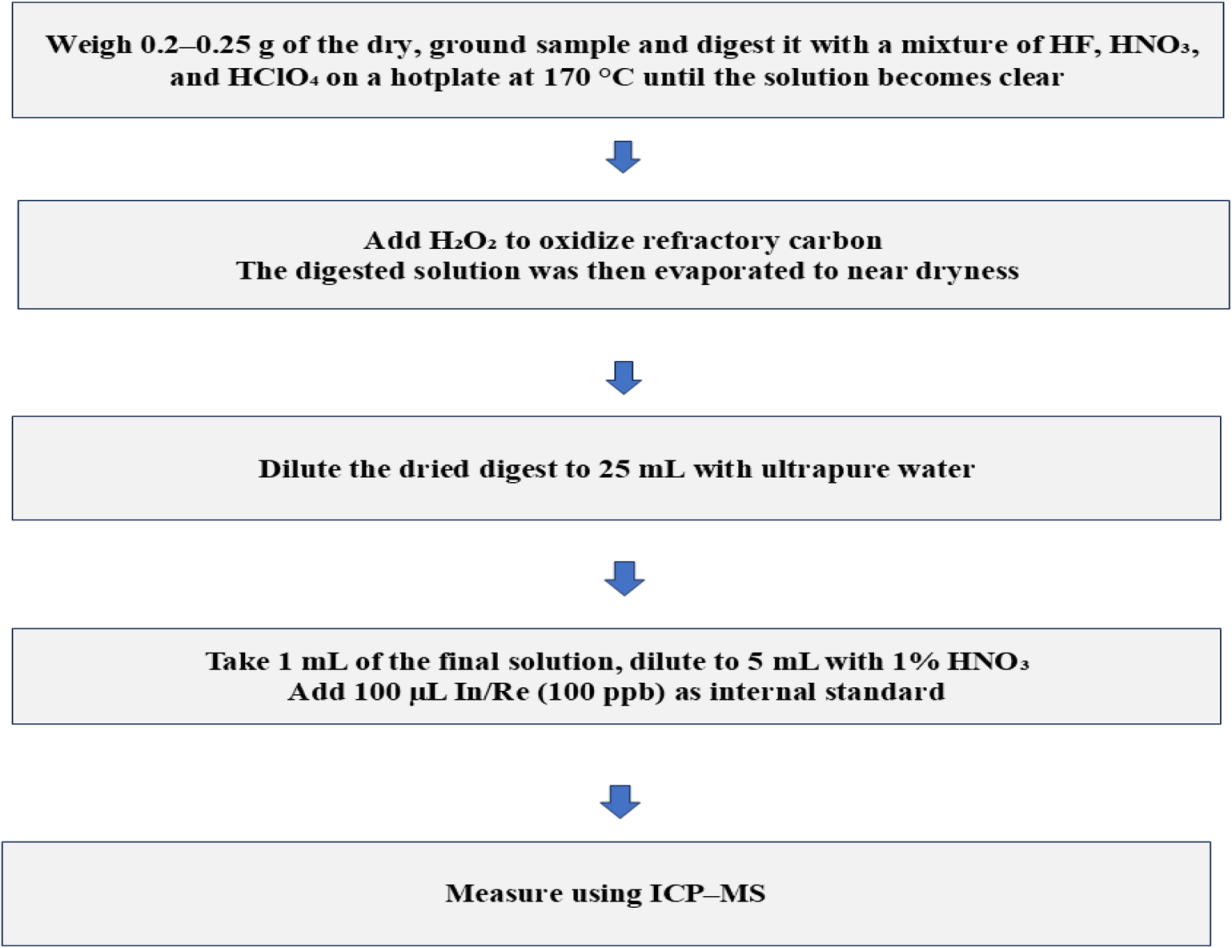
Schematic of the digestion of bottom ash (BA).

For bottom ash (BA), 0.20–0.25 g of dried and ground sample was digested using a mixed-acid system consisting of 5 mL HF (40%), 3 mL HNO_3_ (65%), and 2 mL HClO_4_ (70%) in Teflon digestion vessels. The mixture was heated on a hotplate at 170 °C until the solution became clear. A few drops of H_2_O_2_ were added to ensure complete oxidation of carbonaceous matter. The digested solution was then evaporated to near dryness, leaving approximately 0.5–1 mL of solution, and subsequently diluted to 25 mL with ultrapure water. A total of 1 mL of the final solution was taken for ICP-MS analysis which was further diluted to a total volume of 5 mL with 1% HNO_3_. Each sample was spiked with 100 µL of an internal standard mixture containing indium and rhenium (100 ppb each). Reagent blanks and certified reference materials were treated in the same way as the samples before instrumental measurement, following the procedures outlined in U.S. EPA Method 201A (2020).^28^ This method governs only the sampling procedure; subsequent chemical digestion and analysis of PM_10_ were conducted using the sodium-peroxide fusion protocol described in Section 2.2.1. All analyses were performed in duplicate to account for sample variability. The precision and accuracy of the analytical method were evaluated using reference materials BCR 723 and SRM 2709. Nitric acid (HNO_3_), hydrochloric acid (HCl), perchloric acid (HClO_4_), hydrogen peroxide (H_2_O_2_), and hydrofluoric acid (HF) (Merck, Germany) were used for sample digestion. Working calibration standards for Pd, Pt, Ag, and Au were prepared by diluting single-element stock solutions (1000 mg L^−1^; Milwaukee, WI, USA). Deionized water was obtained from a Milli-Q Plus purification system (Nihon Millipore Kogyo, Tokyo, Japan). Certified reference materials SRM 1633b (NIST, Gaithersburg, MD, USA) and municipal solid waste incineration ash CRM 176 (BCR, Brussels, Belgium) were analyzed for quality assurance. A schematic overview of the digestion procedure for bottom ash (BA) samples is presented in Fig. 4.

### 2.3. ICP-MS analysis and quality assurance

The analysis of quantitative noble metals (Ag, Au, Pt, Pd, and Rh) was performed with an inductively coupled plasma mass spectrometer (ICP-MS, Agilent 7900, USA). Instrumental calibration was performed using multi-element standard solutions prepared at five concentration levels, and linearity was confirmed with *R*^2^ > 0.999 for all analytes. The ICP-MS operating conditions followed the standard procedures recommended in the Agilent 7900 application manual and U.S. EPA Method 6020B. To verify the accuracy of our analytical approach, two reference materials certified as reference materials (BCR 723 – road dust and SRM 2709 – San Joaquin soil) were digested and analyzed through exactly the same method. Recovery yields of Ag, Au, Pt, Pd, and Rh were within 83% and 107%. Recoveries slightly above 100% (maximum 107%) are attributed to small positive analytical biases rather than contamination. Such values can occur when the Na_2_O_2_ fusion achieves more complete dissolution of refractory minerals than the reference method, combined with minor matrix-related effects or instrumental variability at low analyte levels. These deviations are within the acceptable range reported for peroxide-fusion digestion and ICP-MS measurement. The relative standard deviations (RSDs) were generally below 5% for most elements, with slightly higher values (∼8%) observed for Ag and Rh due to their lower concentrations and the known challenges associated with their ICP-MS quantification (see Table S2 in SI).

### 2.4. Scanning electron microscopy and energy-dispersive X-ray spectroscopy (SEM-EDX)

Morphological and microchemical characterization of PM_10_, fly ash, and bottom ash samples was performed using field-emission scanning electron microscopy coupled with energy-dispersive X-ray spectroscopy (SEM-EDX). A JEOL JSM-6700F FE-SEM (Akishima, Tokyo, Japan) equipped with an Oxford Instruments INCA EDX detector was used for all analyses.^31^

Samples were oven-dried at 60 °C, lightly ground, and sieved to <75 µm. A small amount of powder was sprinkled onto conductive carbon adhesive tabs mounted on aluminium stubs. To minimise surface charging under the electron beam, each sample was coated with a ∼10 nm Au/Pd film using a Quorum Q150R ES sputter coater operated at 30 mA for 60 s.

Imaging was conducted under high vacuum using an accelerating voltage of 10–20 kV, probe current of 10–20 µA, and a working distance of 8–10 mm. Secondary electron (SE) images were captured to examine particle morphology, whereas backscattered electron (BSE) images provided compositional contrast for identifying mineral phases and metal-rich particles.

EDX spectra were acquired at 20 kV with a live time of 60 s and a take-off angle of 35°. Quantitative elemental analyses were performed using standardless ZAF corrections. Area maps and point analyses were collected to assess elemental associations, heterogeneity within ash matrices, and the distribution of precious and heavy metals across ash fractions.

Although direct determination of oxidation states using XPS or XRD was not possible in this study due to the lack of local analytical facilities and the very high cost of outsourcing such analyses, the expected chemical forms of the investigated metals can be reliably inferred from previously published work. Silver is typically present as metallic Ag^0^, AgCl, and Ag_2_S in MSWI bottom ash, formed through reactions with chlorides and sulfides during combustion.^8,14^ Gold generally occurs in the metallic state (Au^0^) or as Au–Ag–Cu alloys because of its high chemical inertness and low reactivity under incineration temperatures.^1,13^ Platinum-group elements often exist as metallic nanoparticles or as oxidized species (PtO_*x*_, PdO_*x*_, Rh_2_O_3_), depending on furnace temperature and the presence of aluminosilicate matrices that can encapsulate PGM particles.^32,33^ These oxidation-state patterns have been widely reported across MSWI residues and support the chemical behavior inferred from our concentration and distribution data, even in the absence of direct XPS/XRD measurements.

### 2.5. Statistical analysis and data interpretation

Each experiment was conducted in triplicate. The measured results are presented as mean values ± standard deviation (SD). One-way analysis of variance (ANOVA), principal component analysis (PCA), and Pearson correlation were performed using OriginPro 2019 software (OriginLab Corporation, USA). The graphs were also plotted using OriginPro 2019.

### 2.6. Profitability analysis

This research describes an economic recovery assessment model (ERAM) of three incineration residues – PM_10_, fly ash, and bottom ash – to give a deeper assessment of the potential value of waste-derived products. The economics of potential recovery of precious metals was calculated based on a theoretical calculation model based on the metal concentration calculation using chemical analysis. Two profit scenarios were established; Scenario 1 used recovery efficiency and the market prices of the precious metals present in incineration residues, while Scenario 2 used both operational and capital investment costs for the precious metal recovery. Economic results from such models can be useful in developing optimal treatment and recovery strategies for incineration residues, as well as give a realistic estimate of the recoverability and profitability of precious metals recovery. Scenario 1 was calculated based on the annual emissions (AE) of each incinerator, derived from ash generation, flue gas flow rate, and actual operational capacity (Table S1 in SI). In this scenario, the economic value was estimated using the recovery efficiencies reported for the Hinwil plant in Switzerland (Ag: 57.4%; Au: 28.8%; for both Pt and Pd: 30%)^9,14^ and the 2025 market prices of the respective metals (Ag: 0.98 EUR per g; Au: 81.81 EUR per g; Pt: 30.53 EUR per g; Pd: 29.6 EUR per g; Rh: 212 EUR per g).^34^ Operational and investment costs were not included in this scenario. The calculation formulas were adopted from Nguyen *et al.* (2021),^35^ Nguyen *et al.* (2022),^36^ Mehr *et al.* (2021),^9^ and Chuchro *et al.* (2025).^14^

• The annual emission of precious metals (AE, g per year) was determined using the following equations:

1 AE_ash (BA, FA)_ = *C*_metal (BA, FA)_ × GR_ash (BA, FA)_ × *P* × AOT

2 AE_PM10_ = *C*_PM10_ × *F* × AOT

where: *C*_ash_ is the concentration of each precious metal (mg kg^−1^) in fly ash (FA) or bottom ash (BA); GR_ash (BA, FA)_ is the amount of ash or slag produced (kg ton^−1^ waste); *F* is the flue gas flow rate (Nm^3^ h^−1^); *P* is the incineration capacity (ton h^−1^); and AOT is the average annual operating time (h per year);

• The economic value of recovered metals (*H*_metal_, EUR per year) was estimated as:3
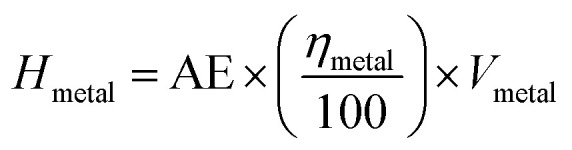
where: AE is the annual metal emission (g per year), *η*_metal_ (%) is the recovery efficiency (%) based on the Hinwil model, *V*_metal_ is the market price of each metal (EUR per g) in 2025, *H*_metal_: economic value of recovered metals (EUR per year).

Scenario 2 estimated the net profit by including both operational expenditures (OPEX) and investment depreciation (CAPEX). In recent years, the Hinwil-Q-Metal plant (Switzerland) has been considered the world's leading advanced model in recovering metals from municipal solid waste incinerator bottom ash (MSWI bottom ash). The plant applies a dry treatment process combined with sizing, magnetic separation, eddy current separation, and sensor-based separation technologies to effectively recover iron, non-ferrous metals (Al, Cu, Zn, Pb), and even precious metals such as Ag and Au, without using energy-consuming and chemical-intensive hydrometallurgical or thermal treatment processes. The success of Hinwil-Q-Metal has proven that MSWI bottom ash is not only a waste that needs to be treated but also a valuable secondary resource, and at the same time creates an important premise for studies to evaluate the possibility of recovering precious metals from bottom ash in developing countries, including Vietnam.^4,9^

The model parameters were adapted from the Hinwil facility.^9,14^ OPEX included electricity consumption (8.28 kWh per ton ash) at an electricity price of 0.1291 EUR per kWh, maintenance costs (1.25% CAPEX for years 1–2 and 2.5% CAPEX for years 3–20), labor costs (80 000 EUR per year), and chemical and analytical expenses (25 000 EUR per year). The initial plant construction cost is dependent on plant capacity. It is estimated that the investment depreciation (CAPEX) cost for constructing a plant with a capacity of $50 000 tons per year is approximate 3.447 million EUR. Average emission flow rate of Hinwill: *F* = 87 000 Nm^3^ h^−1^; The relevant formulas are as follows:

• Economic value of precious metals based on the Hinwil–Qmetal model (EUR per year):4
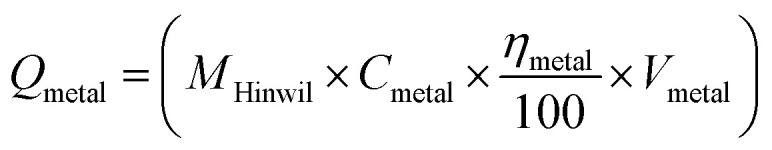
where: *Q*_metal_ = annual economic value of the metal; *M*_Hinwil_ = annual mass of MSWI residues in the Hinwil model; *C*_metal_ = concentration of the precious metal (consistent notation); *η*_metal_ = recovery efficiency (%) and *V*_metal_ = market value of the metal. Where *M*_Hinwil_ represents the estimated throughput for a 50 000 ton per year facility.

• Annual net profit (EUR per year):

5 ANCF = *Q*_metal_ – (OPEX + *C*_CAPEX_)

6 OPEX = *V*_1_ + *V*_2_ + *V*_3_ + *V*_4_

where *V*_1_–*V*_4_: denote the costs of electricity, maintenance, labor, and chemicals/analysis, respectively. Investment depreciation over 20 years (*N* = 20) was computed as:

*C*_CAPEX_ refers to the assumed investment depreciation cost over a 3–20 year period (*N* = 20).7
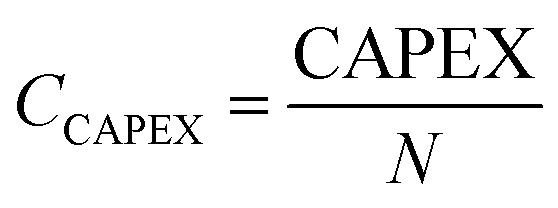


In Scenario 2, the economic assessment was based on the process configuration of the existing Hinwil MSWI residue treatment facility in Switzerland. The plant integrates a multi-step metal recovery system, which includes: (i) mechanical pretreatment (drying, sieving, magnetic separation, eddy-current separation) to concentrate metal-rich fractions; (ii) further density and sensor-based sorting to isolate precious-metal-bearing streams; (iii) hydrometallurgical leaching using acidic chloride media to dissolve Au, Ag, Pt, Pd, and Rh; and (iv) selective metal recovery through precipitation, adsorption, or electrochemical deposition. The facility is fully automated and equipped with continuous conveyors, magnetic and eddy-current separators, leaching reactors, filtration units, and metal-recovery cells. This workflow ensures both high recovery efficiency and stable operational throughput, and the associated energy, labor, and maintenance requirements were incorporated into the economic model applied in Scenario 2.

## 3. Result and discussion

### 3.1. Surface characterization of incineration residues

**Fig. 5:**
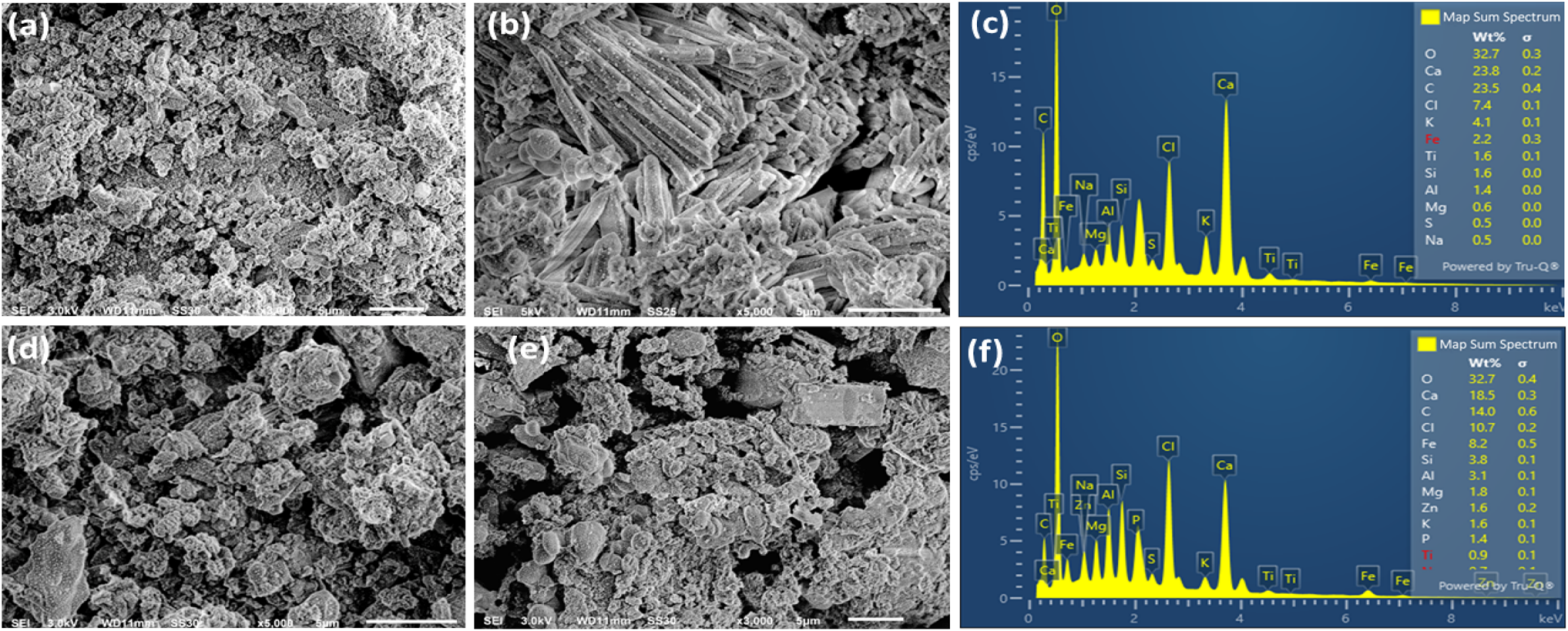
SEM and EDX images of fly ash (a–c) and bottom ash (d–f) obtained from municipal solid waste incineration residues.

The surface morphology and elemental composition of fly ash and bottom ash were analyzed using SEM and EDX (Fig. 5). The SEM images of fly ash (Fig. 5a and b) showed fine particles containing glassy spherical grains together with needle- or plate-like crystalline structures. Such spherical and glassy morphologies are characteristic of amorphous silicate phases formed under high-temperature combustion and rapid cooling, as widely reported for MSWI fly ash.^37,38^ The needle- or plate-like structures observed here are consistent with Ca–Al–Si minerals such as anorthite or gehlenite, which commonly crystallize during incineration processes.^39,40^ The smooth and homogeneous surfaces further indicated the presence of a dominant glassy matrix encapsulating mineral and metallic components.^18^

For bottom ash (Fig. 4d and e), the SEM images revealed angular grains, larger particle sizes, and rough, porous surfaces with cracks and few spherical grains. This morphology is typical of BA and reflects mechanical fragmentation, incomplete combustion, and partial melting of heterogeneous feedstock, as previously documented.^41^ The porous texture and fractured structures have been reported to facilitate the surface deposition or aggregation of metallic particles, including precious metals or metal alloys.^13^

EDX was performed to analyze the elemental composition of FA (Fig. 4c) and BA (Fig. 2f). For FA, the primary elements identified were O (32.7%), Ca (23.8%), C (23.5%), K (7.4%), Cl (4.1%), Fe (2.2%), and Si (1.6%) (Fig. 4c). The high CaO content and the presence of Cl indicated the existence of Ca–Cl–K phases in the fly ash. These elements originated from inorganic waste components such as salts, plastics, and lime. The relatively low Si and Al concentrations compared with coal combustion fly ash showed that the two had different compositions. In contrast, coal-combustion fly ash typically contains much higher proportions of Si and Al due to its dominant silicate–aluminate mineralogy, with common oxide compositions of approximately SiO_2_ (40–60%), Al_2_O_3_ (20–30%), Fe_2_O_3_ (4–10%), and CaO (1–5%).^42,43^ Therefore, the relatively low Si and Al concentrations observed in our MSWI fly ash clearly distinguish its composition from that of coal-derived fly ash.

Similarly, EDX analysis of bottom ash revealed similar major elements, including O (32.7%), Ca (18.5%), Cl (14.0%), C (13.0%), Fe (8.8%), Si (3.8%), and Al (3.1%) (Fig. 2f). The higher Fe and Cl contents in BA in comparison with FA suggested the presence of heavy metal salts and/or oxides in this sample.

In summary, SEM-EDX analysis indicated obvious structural and compositional differences between fly ash and bottom ash. These differences strongly affected the occurrence and distribution of precious metals (Au, Ag, Pt, Pd, and Rh). Fly ash's dense glassy silicate–aluminate matrix and spherical morphology tend to embed precious metals within crystal lattices or glass phases, resulting in less accessible and more dispersed forms. In contrast, the porous/angulated characteristics of the bottom ash facilitated the occurrence of precious metals as free particles or metal alloys. These microstructural features were deemed significant to select appropriate recovery and treatment strategies for precious metals from incineration residues.

### 3.2. Evaluation of precious metal contents in fly ash, bottom ash, and fine particulate matter (PM_10_) from MSW incinerators

**Fig. 6:**
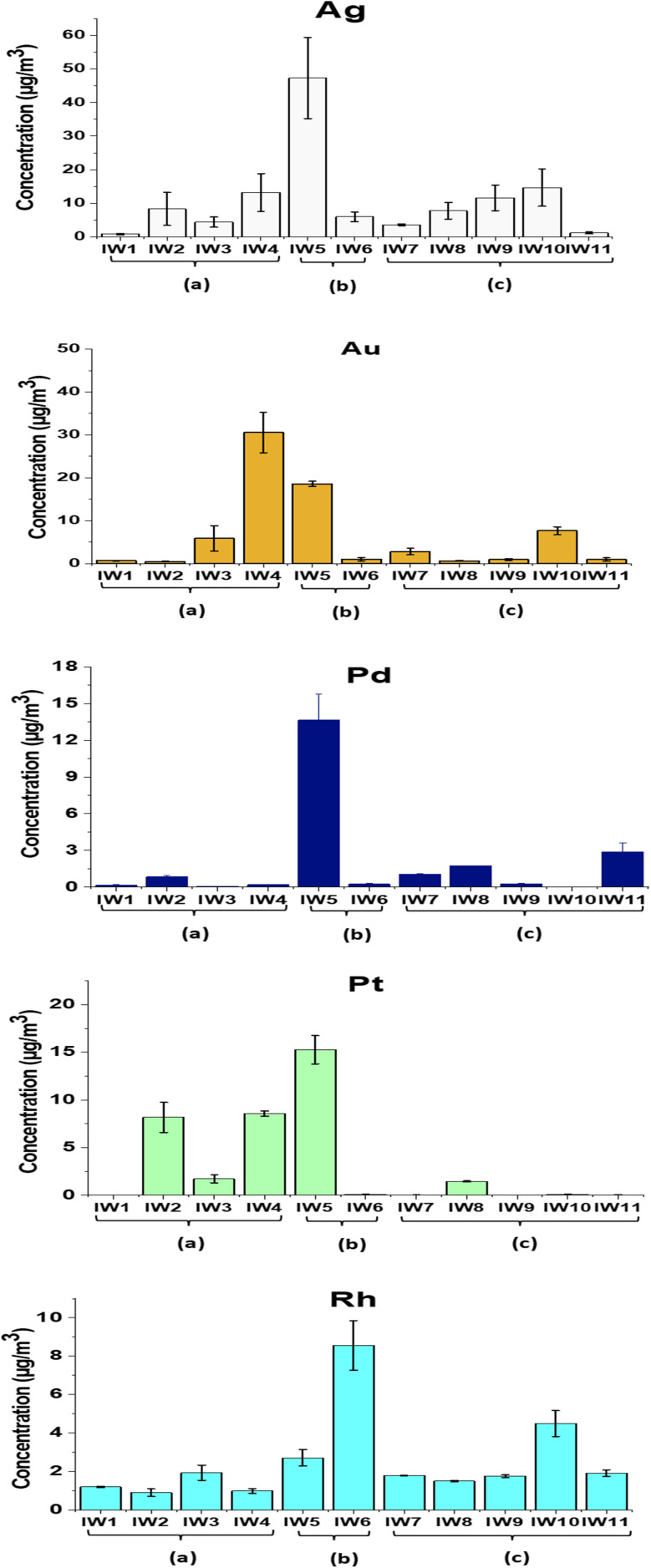
Concentrations of precious metals (Ag, Au, Pd, Pt and Rh) in PM_10_, from municipal solid waste incinerators. (a) Municipal wastes (100%); (b) industrial wastes/municipal wastes (70%/30%); (c): industrial wastes (100%).

**Fig. 7:**
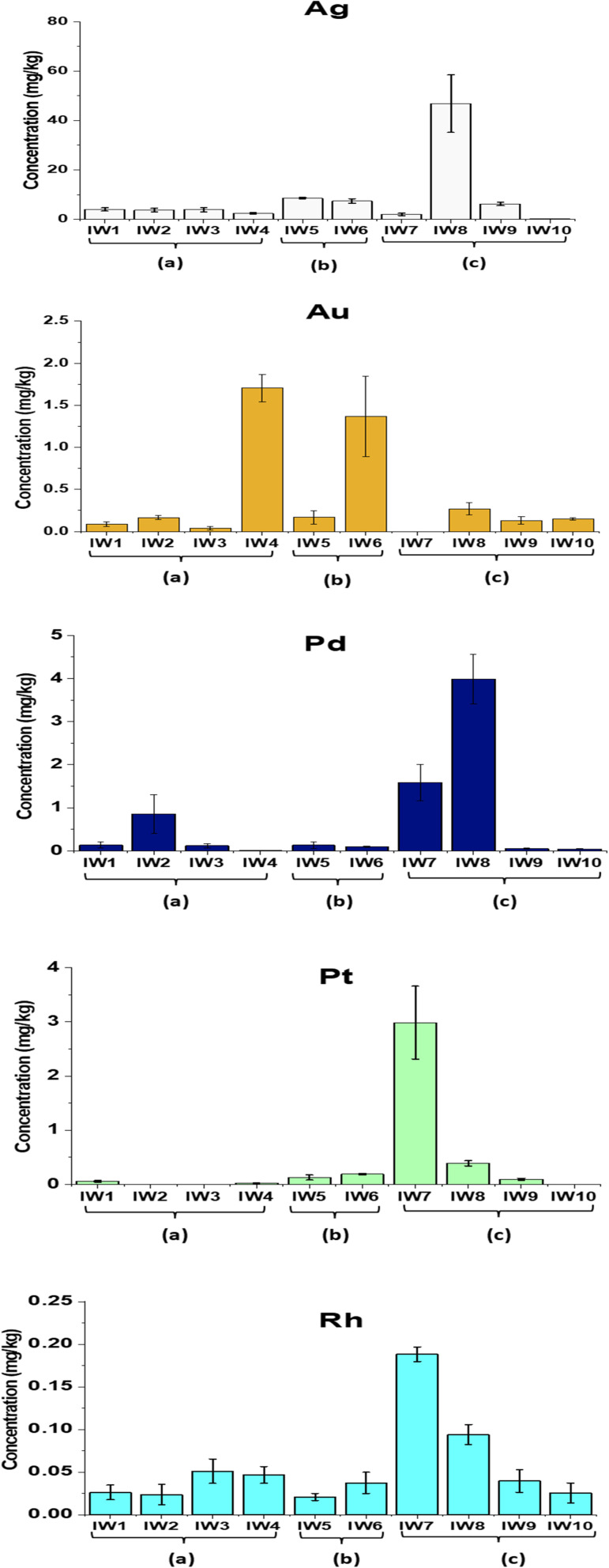
Concentrations of precious metals (Ag, Au, Pd, Pt and Rh) in fly ash from municipal solid waste incinerators. (a) Municipal wastes (100%); (b) industrial wastes/municipal wastes (70%/30%); (c): industrial wastes (100%).

**Fig. 8:**
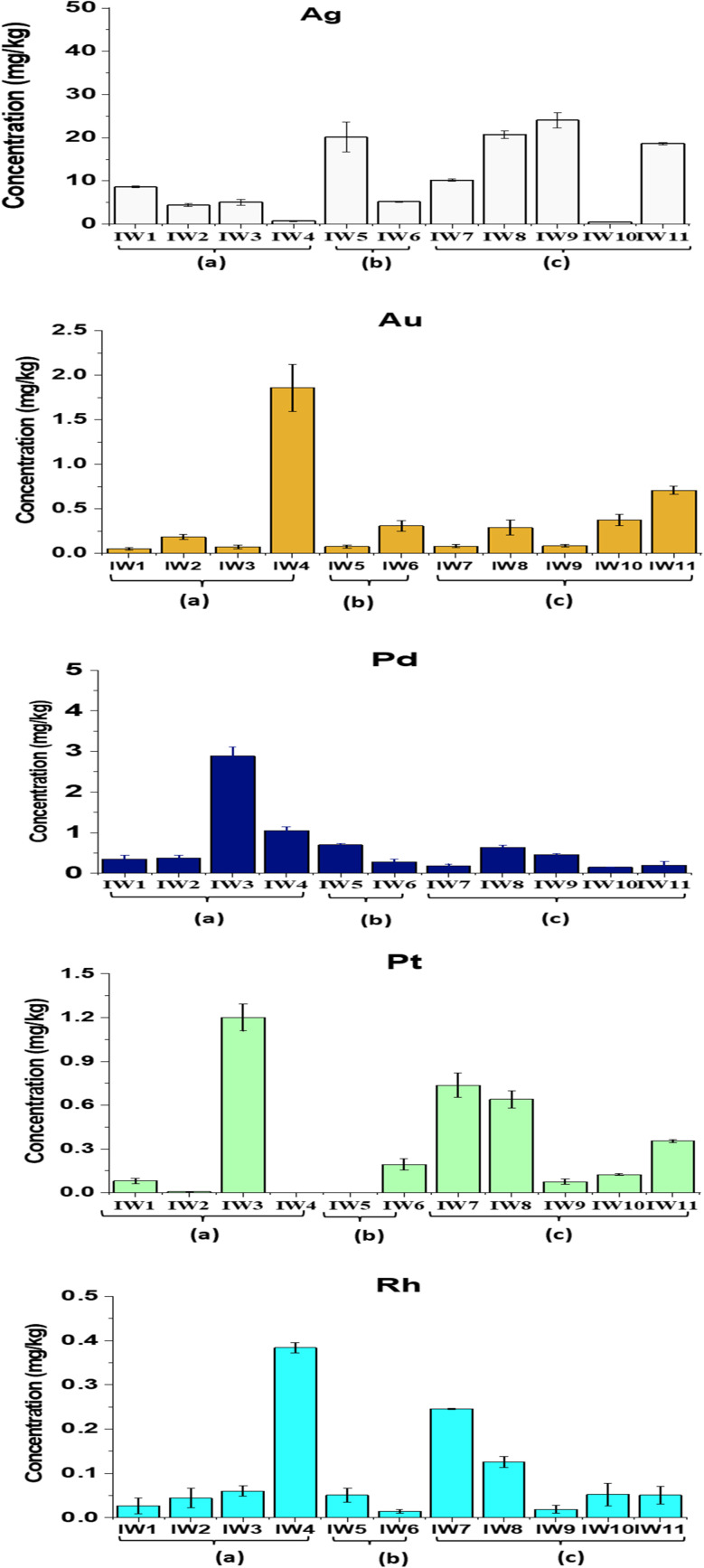
Concentrations of precious metals (Ag, Au, Pd, Pt and Rh) in bottom ash from municipal solid waste incinerators. (a) Municipal wastes (100%); (b) industrial wastes/municipal wastes (70%/30%); (c): industrial wastes (100%).

The analytical procedure showed robust performance, with recovery yields for Ag, Au, Pt, Pd, and Rh ranging from 83% to 107%. The relative standard deviations (RSDs) were generally below 5% for most elements, with slightly higher values (∼8%) observed for Ag and Rh due to their lower concentrations and the known challenges associated with their ICP-MS quantification (see Table S2 in SI). The concentrations of five precious metals (Ag, Au, Pt, Pd, and Rh) in incineration residues obtained from three types of waste feedstocks, (a) municipal solid waste, (b) mixed municipal-industrial waste, and (c) industrial waste, are presented in Table 1 and Fig. 6–8.

**Table 1:** Mean concentrations (±SD) of precious metals in PM_10_, fly ash (FA), and bottom ash (BA) from the investigated MSW incineration plant^a^

Incinerators	Ag	SD (Ag)	Au	SD (Au)	Pt	SD (Pt)	Pd	SD (Pd)	Rh	SD (Rh)
**PM10 – particulate matter (µg Nm** ^**−3**^ **)**										
Municipals wastes										
IW1	0.829	0.192	0.727	0.065	nd	nd	0.153	0.047	1.19	0.036
IW2	8.32	4.92	0.515	0.054	8.17	1.16	0.843	0.131	0.887	0.195
IW3	4.35	1.508	5.88	2.96	1.72	0.420	0.062	0.0073	1.93	0.395
IW4	13.2	5.66	30.6	4.70	8.57	0.279	0.199	0.0086	0.981	0.121
Industrial waste (70%) + municipals wastes (30%)										
IW5	47.3	12.1	18.6	0.616	15.3	1.50	13.6	2.14	2.70	0.423
IW6	5.97	1.45	1.06	0.411	0.105	0.014	0.241	0.066	8.56	1.29
Industrial waste (100%)										
IW7	3.59	0.254	2.85	0.771	0.045	0.006	1.07	0.026	1.78	0.009
IW8	7.75	2.50	0.648	0.080	1.45	0.062	1.75	0.0092	1.49	0.041
IW9	11.6	3.84	0.950	0.214	nd	nd	0.282	0.0164	1.75	0.071
IW10	14.7	5.56	7.66	0.958	0.093	0.015	0.031	0.0062	4.48	0.674
IW11	1.19	0.303	0.990	0.380	0.058	0.017	2.89	0.708	1.91	0.166
Mean	10.8 ± 13.0	3.5 ± 3.5	6.40 ± 9.7	1.02 ± 1.5	3.23 ± 5.5	1.26 ± 0.6	1.92 ± 4.0	0.288 ± 0.6	2.52 ± 2.2	0.311 ± 0.4

**FA – fly ash (mg kg** ^**−1**^ **)**										
Municipals wastes										
IW1	4.02	0.698	0.086	0.028	0.053	0.013	0.133	0.070	0.026	0.009
IW2	3.74	0.735	0.166	0.024	nd	nd	0.857	0.447	0.024	0.012
IW3	3.86	0.865	0.038	0.019	nd	nd	0.119	0.053	0.051	0.014
IW4	2.41	0.333	1.70	0.166	0.019	0.006	0.009	0.005	0.047	0.010
Industrial waste (70%) + municipals wastes (30%)										
IW5	8.55	0.298	0.167	0.076	0.125	0.047	0.132	0.084	0.021	0.004
IW6	7.41	0.927	1.37	0.480	0.187	0.014	0.093	0.018	0.037	0.012
Industrial waste (100%)										
IW7	1.99	0.479	nd	nd	2.99	0.674	1.59	0.423	0.188	0.009
IW8	46.8	11.7	0.270	0.074	0.387	0.048	3.99	0.574	0.094	0.012
IW9	6.24	0.725	0.132	0.042	0.092	0.019	0.053	0.018	0.039	0.013
IW10	0.162	0.014	0.149	0.012	nd	nd	0.047	0.008	0.026	0.012
Mean	8.52 ± 14.4	1.67 ± 3.7	0.453 ± 0.6	0.102 ± 0.2	0.550 ± 1.2	0.117 ± 0.3	0.702 ± 1.3	0.170 ± 0.2	0.055 ± 0.1	0.011 ± 0.0

**BA – bottom ash (mg kg** ^**−1**^ **)**										
Municipals wastes										
IW1	8.59	0.179	0.054	0.014	0.081	0.018	0.351	0.090	0.027	0.018
IW2	4.38	0.308	0.184	0.027	0.007	0.004	0.380	0.063	0.044	0.022
IW3	5.04	0.635	0.072	0.021	1.20	0.093	2.89	0.216	0.060	0.012
IW4	0.733	0.127	1.86	0.264	nd	nd	1.05	0.090	0.384	0.012
Industrial waste (70%) + municipals wastes (30%)										
IW5	20.1	3.45	0.076	0.017	nd	nd	0.698	0.035	0.051	nd
IW6	5.17	0.116	0.308	0.058	0.194	0.038	0.282	0.059	0.015	1.142
Industrial waste (100%)										
IW7	10.2	0.275	0.082	0.019	0.736	0.084	0.187	0.042	0.245	0.002
IW8	20.7	0.856	0.289	0.086	0.641	0.059	0.641	0.055	0.126	0.013
IW9	24.0	1.77	0.086	0.016	0.076	0.018	0.457	0.025	0.019	0.010
IW10	0.500	0.110	0.375	0.062	0.125	0.005	0.150	0.003	0.050	0.007
IW11	18.6	0.289	0.709	0.045	0.354	0.010	0.202	0.092	0.051	0.020
Mean	10.7 ± 9.3	0.738 ± 1.1	0.372 ± 0.6	0.07 ± 0.1	0.311 ± 0.4	0.030 ± 0.0	0.663 ± 0.9	0.070 ± 0.1	0.097 ± 0.1	0.114 ± 0.4

a nd: “not detection”; SD: standard deviation; replication *n* = 3.

The average concentrations of these metals in fine particulate matter (PM_10_) from different incinerators ranged from 0.311 to 10.8 µg Nm^−3^. The metals followed the decreasing order of abundance: Ag > Au > Pt > Rh > Pd. In the groups burning municipal and mixed wastes, Ag exhibited notably high concentrations, reaching 47.3 µg Nm^−3^ in IW5, while Au also showed elevated levels, ranging from 5.88 µg Nm^−3^ in IW3 to 30.6 µg Nm^−3^ in IW4. The highest concentrations of Pt, Pd, and Rh were found in IW4 (Pt: 8.57 µg Nm^−3^), IW5 (Pt: 15.3 µg Nm^−3^; Pd: 13.6 µg Nm^−3^), and IW6 (Rh: 8.56 µg Nm^−3^), respectively (ANOVA, *p* < 0.05). These differences were statistically significant based on one-way ANOVA (*p* < 0.05), with the full ANOVA results provided in the SI (Table S4 in SI).

Few studies had investigated the concentrations of precious metals in incinerator flue gases, as most previous research focused on fly ash and bottom ash. Therefore, it was difficult to establish direct comparisons or accurately assess the emission sources of these metals in gaseous samples. However, when compared with airborne dust samples from road environments, the concentrations of Pd, Pt, and Rh in this study were considerably higher than those reported in road dust aerosols from Rome (21.2–85.7, 7.8–38.8, and 2.2–5.8 pg m^−3^ for Pd, Pt, and Rh, respectively),^44^ from Germany (Pd: 0.20–14.6 pg m^−3^),^45^ in PM_2_._5_ from Mexico (Pt: 38.4 pg m^−3^),^46^ and in PM_10_ and PM_2.5_ from India (Pt: 0.86–12.3 ng m^−3^; Pd: 2.7–111 ng m^−3^).^47^ The concentrations of Pd, Pt, and Rh measured in this study were noticeably higher than those reported in other cities and countries. This difference may reflect several region-specific factors, including (i) increasing emissions from vehicular catalytic converters associated with growing traffic density, (ii) differences in waste composition such as a higher proportion of metal-rich consumer products and electronic waste entering the municipal waste stream, and (iii) operational conditions of the local MSWI plants, which may enhance the partitioning of PGEs into the ash fraction. These elevated concentrations suggest that MSWI residues in the studied area may constitute a comparatively richer secondary resource for platinum-group metals, thereby indicating promising potential for future recovery and circular-economy applications.

The mean concentrations of the studied precious metals in fly ash followed the order Ag (8.52 mg kg^−1^) > Pd (0.702 mg kg^−1^) > Pt (0.550 mg kg^−1^) > Au (0.453 mg kg^−1^) > Rh (0.055 mg kg^−1^) (ANOVA, *p* < 0.05). These values were calculated from all FA samples (*n* = 11) across municipal, mixed, and industrial incinerators, as shown in Table 1. Ag tended to accumulate more in the industrial waste group (c), with the highest level reaching 46.8 mg kg^−1^, significantly higher than in the municipal (a) and mixed (b) waste groups. This trend was consistent with the widespread use of Ag in the electronics, chemical, and reflective materials industries. In contrast, Au concentrations were higher in the municipal and mixed waste groups, with maximum values of 1.70 mg kg^−1^ (a) and 1.37 mg kg^−1^ (b), respectively. This pattern reflected the contribution of household electronic devices, jewelry, and cosmetic waste as potential sources of Au. The platinum-group metals (Pt, Pd, and Rh) mainly accumulated in industrial and mixed wastes. The highest concentrations of Pd, Pt, and Rh in industrial waste ashes were 3.99, 2.99, and 0.188 mg kg^−1^, respectively. The high levels of Pt and Pd were likely related to electronic waste inputs.^44^ Some studies also reported that road dust contained high amounts of these metals due to vehicle emissions.^8^ Pt, Pd, and Rh mainly came from automotive catalytic converters installed in exhaust systems.^8,45^ This suggested that part of these metals could have entered the waste stream during waste collection.

Overall, the concentrations of precious metals in fly ash observed in this study were higher than those reported previously. The Ag content in industrial waste ash was considerably higher than the 5–20 mg kg^−1^ range reported by Morf *et al.* (2013) for MSWI fly ash in Switzerland.^8^ Similarly, Au concentrations in the fly ash from municipal and mixed waste incinerators in this study exceeded the 0.2–0.8 mg kg^−1^ range reported by Muchova *et al.* (2009) for Dutch fly ash^13^ and the 0.58 mg kg^−1^ reported for northern Italian incinerators.^32^ The Pd, Pt, and Rh concentrations in industrial waste ashes also surpassed those reported by Birloaga *et al.* (2013) (Pd < 1 mg kg^−1^ in European MSWI residues)^32^ and Song *et al.* (2015) (Pt: 0.1–1 mg kg^−1^; Rh: 0.05–0.1 mg kg^−1^ in Chinese fly ash).^33^

Precious metal concentrations in bottom ash were generally of greater interest due to the higher recovery potential compared with fly ash. Among all bottom ash samples, Ag exhibited the highest concentrations relative to the other metals. Among the municipal wastes (a), Ag concentration ranged from 0.733 to 8.59 mg kg^−1^, and for mixed wastes (b) and industrial wastes (c) groups, the values were significantly higher, ranging from 5.17 to 20.1 mg kg^−1^ and 0.50 to 24.0 mg kg^−1^, respectively. The Ag content in industrial waste in this study was higher than the value reported by Chuchro *et al.* (2025) for European bottom ash (20.1 mg kg^−1^)^14^ and was comparable to the average Ag concentration of 11 mg kg^−1^ reported by Beikmohammadi *et al.* (2023) in Tehran.^1^

In comparison to Ag, Au, Pt, Pd, and Rh levels between samples were highly variable. The mean value of Au was 0.738 mg kg^−1^, while the highest content of Au occurred in municipal waste group (a) with 1.86 mg per kg (IW4), which could be attributed to jewelry or light electronic items in the municipal waste. These values were generally consistent with the earlier reports of Chuchro *et al.* (2025)^14^ and Beikmohammadi *et al.* (2023),^1^ which reported Au at around 0.3 mg kg^−1^. Pd and Pt averaged 0.311 mg kg^−1^ and 0.663 mg kg^−1^, respectively; but sample IW3 contained anomalously elevated concentrations (Pd: 3.0 mg kg^−1^, Pt: 1.20 mg kg^−1^), possibly derived from auto catalysts or specific industrial waste inputs.

These results agreed with previous findings in Japan,^24^ where Pt levels in petroleum waste incinerators were higher (0.083–0.392 mg kg^−1^), middle in solid waste incinerators (0.212–0.301 mg kg^−1^), and lowest in municipal waste incinerators (0.0609–0.0737 mg kg^−1^). Rh had the lowest levels among the metals of interest, averaging 0.097 mg kg^−1^. Other studies also reported very low Rh levels in ash due to the fact that it is largely used in automobile catalysts and produces oxides, disperses or volatilizes when incinerated.^48^ The measured concentrations of all precious metals in this study exceeded the average crustal abundances of these elements: Ag (70 ppb, ≈0.07 mg kg^−1^), Au (2.5 ppb, ≈0.0025 mg kg^−1^), Pd (0.4 ppb, ≈4 × 10^−4^ mg kg^−1^), Pt (0.4 ppb, ≈4 × 10^−4^ mg kg^−1^), and Rh (0.06 ppb, ≈6 × 10^−5^ mg kg^−1^).^15^

Overall, Ag dominated across all three waste types, with the highest prevalence in industrial waste (likely related to electronic components, chemicals, and cosmetics), followed by mixed waste and municipal waste. Conversely, Au was more abundant in municipal and mixed waste. This reflected its sources from discarded electronics and jewelry. Pt, Pd, and Rh were usually more concentrated in industrial waste than in municipal waste. This was consistent with their direct use in industrial processes, electronics, chemical manufacturing, and biomedical applications.^49^

### 3.3. Distribution characteristics of precious metals in incinerator samples

**Fig. 9:**
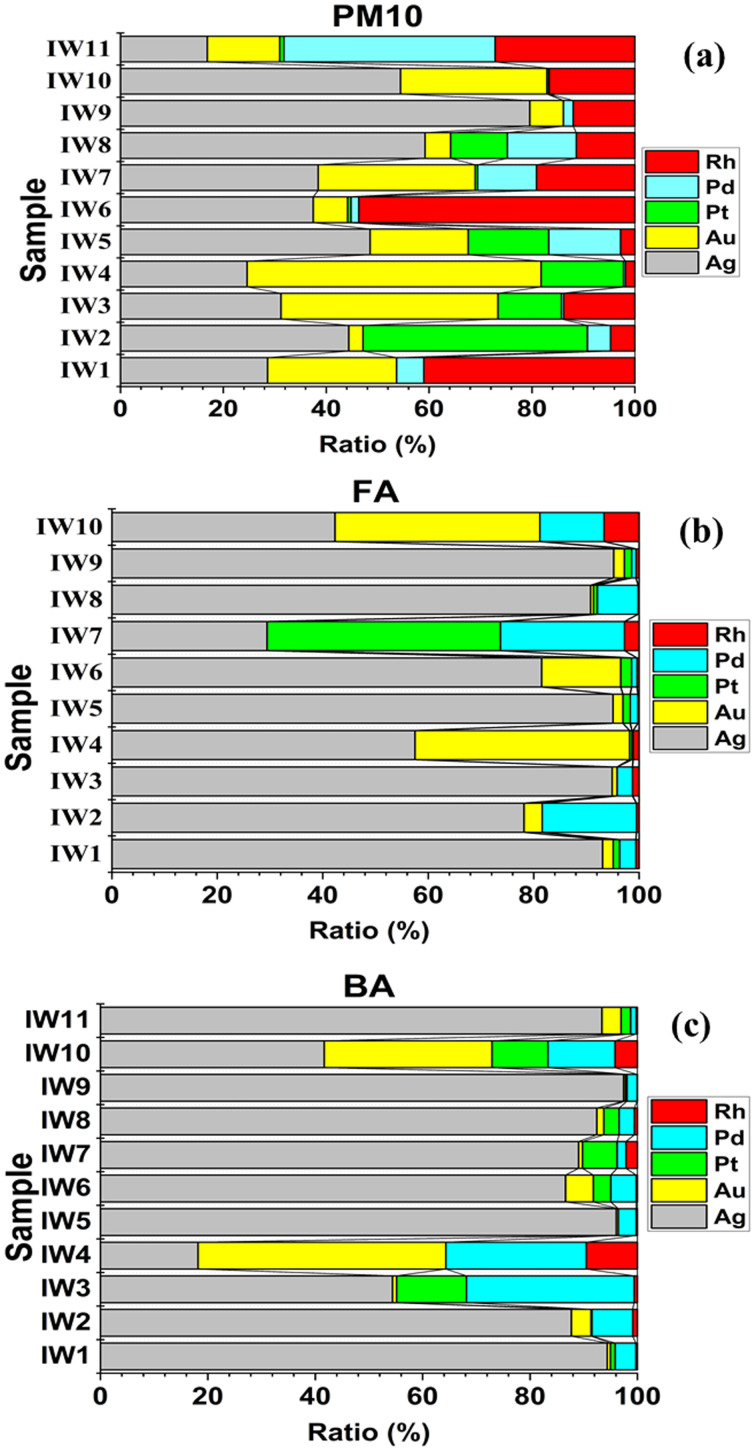
Percentage distribution of target metals (Ag, Au, Pt, Pd, and Rh) across fine particulate matter (PM_10_) (a), fly ash (b), and bottom ash (c) generated from municipal solid waste incineration.

Five target metals (Ag, Au, Pt, Pd, and Rh) distribution in three incinerator residues: fine particulate matter (PM_10_), fly ash, and bottom ash, was assessed. Results are shown in Fig. 9 and Table S3 in SI.

In PM_10_, noble metals were heterogeneously distributed and varied extensively from sample to sample (Fig. 9a). Ag, Au, Pd, and Rh were dominant in different samples by relative percentages of 17–80%, 3–57%, 0–44%, 0–41%, and 2–54%, respectively (see Table S3 (SI)). Au was significantly enriched in IW4 (57%) and IW10 (28%), whereas Ag was dominant in IW9 (80%), IW8 (59%), IW5 (49%), and IW2 (44%). A few samples had high concentrations of Rh, namely IW6 (54%) and IW1 (41%), while Pd reached a peak at IW11 (41%) and, overall, accounted for 10–20% in the rest of the samples. Pt hardly took the lead, though, in IW2 (44%), it appeared at a comparable level to Ag. These modes replicated the formation mechanisms of PM_10_, with high-surface-area particles acting to capture volatile or re-condensed metal upon cooling of flue gas.

In fly ash, the percentage distribution shows a very clear trend: Ag dominates over most of the other metals, accounting for up to 95% of the total precious metals (Fig. 9b). The remaining elements (Au, Pd, Rh, Pt) appear only in minor proportions, except in sample IW7, where Pt and Pd contribute more substantially. This pattern is consistent with the characteristics of fly ash, fine, lightweight particles with high surface area, that facilitate the condensation and adsorption of Ag from the gas phase.

The distribution ratio of Ag in bottom ash remains the highest in most samples, ranging from 42–97% (Fig. 9c), with a rare exception observed in sample IW4 (Ag: 18%). In contrast, Au and Pd exhibit the highest proportions of 46% and 26%, respectively. The remaining metals (Pt and Rh) generally constitute less than 10% in all samples. These results reflect the nature of bottom ash, where less volatile metals and those associated with solid mineral phases tend to be retained, and Ag in particular exhibits a strong affinity for binding within the mineral matrix of the slag.

The percentage distribution highlights a distinct partitioning of precious metals according to their volatility and waste origin. More volatile elements (Ag, Au, Pd) are mainly concentrated in PM_10_ and fly ash, whereas thermally stable elements (Pt, Rh) accumulate in bottom ash. Differences between municipal, mixed, and industrial waste streams indicate that feedstock composition plays a decisive role in governing the distribution patterns of precious metals. Industrial waste (IW7–IW11) is the primary source of Pt, Pd, and Rh, metals typically associated with automotive catalysts and the electronics sector. In contrast, municipal and mixed waste streams are richer in Ag and Au, reflecting contributions from household electronics and consumer products.

The percentage distributions observed in this study differ from several previously reported datasets on precious metals in municipal solid waste, including Muchova & Bakker (2009) (Ag: 5–10%; Au: 3–6%; Pd: 60–70%),^13^ Morf *et al.* (2013) (Ag: 10%; Au: 8%; Pt: 15%; Pd: 60%; Rh: 7%),^8^ and Chuchro *et al.* (2025) (Ag: 6%; Pd: 72%).^14^ These differences underscore that the precious-metal composition is strongly influenced by the characteristics of the input waste and the incineration technology (temperature, gas recirculation, ash recovery).

On the whole, the results demonstrated strong dependencies of metal distribution on waste type, particle morphology, and further development under incineration processes overall. The heterogeneity observed warranted the need for more detailed recovery strategies for the different residue types, depending on metals' state and concentration.

### 3.4. Multivariate regression analysis and correlation assessment of precious metals in waste incinerators

**Fig. 10:**
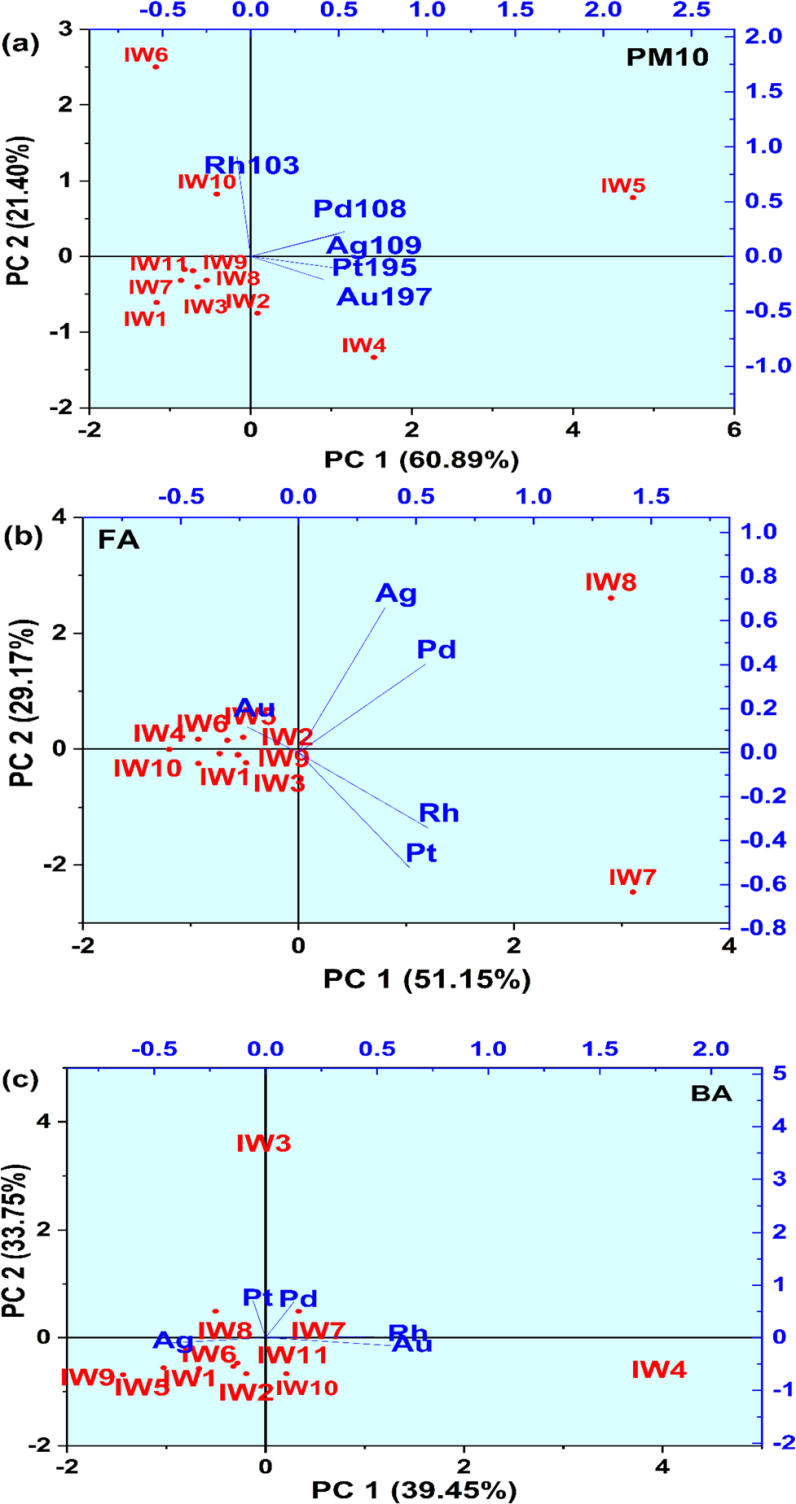
Principal component analysis (PCA) of precious metals in PM_10_ (a), fly ash (b), and bottom ash (c).

**Fig. 11:**
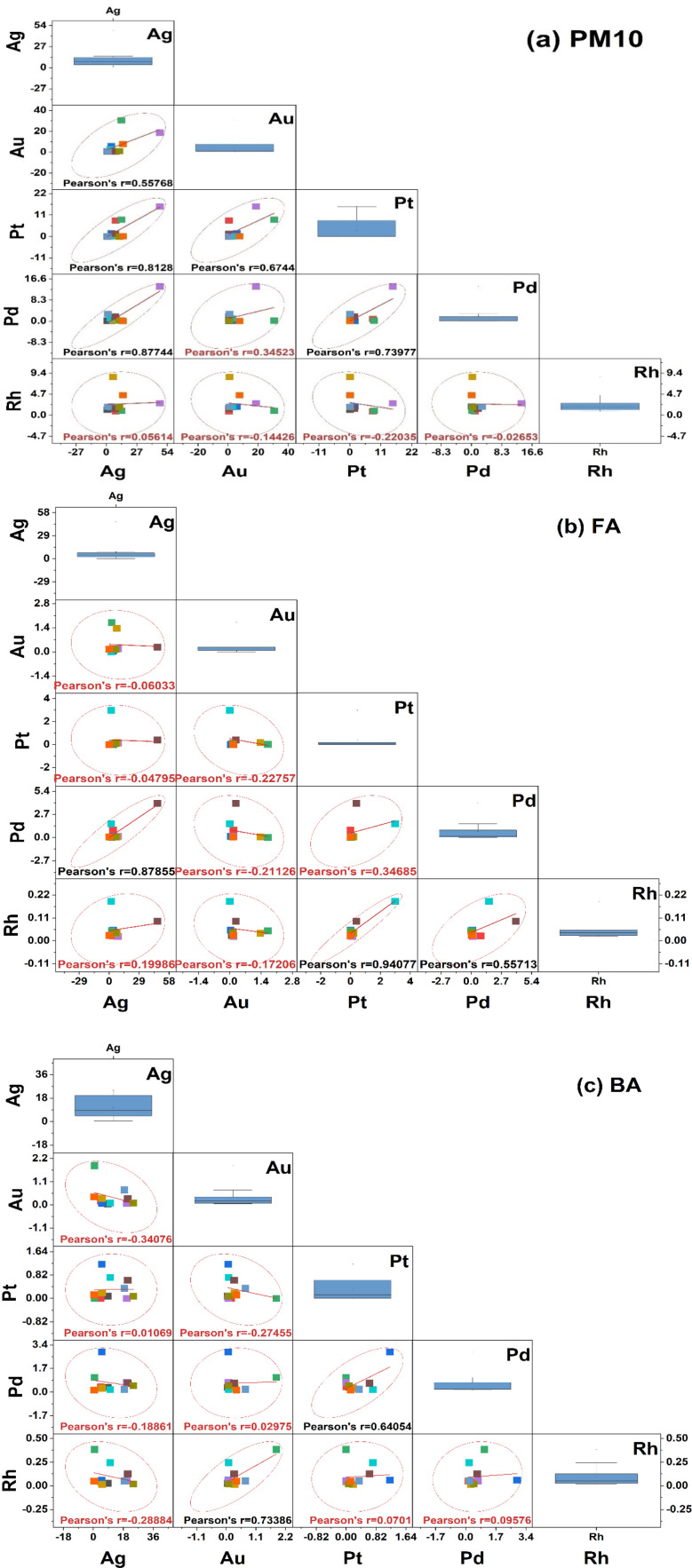
Pearson correlation analysis of precious metals in incineration residues, in PM_10_ (a), fly ash (b), and bottom ash (c).

Principal component analysis (PCA) and Pearson correlation analysis were employed to investigate the distribution patterns and potential emission sources of precious metals across all three residue types, PM_10_, fly ash (FA), and bottom ash (BA). The results are shown in Fig. 10 and 11 and indicated that the metals were from divergent sources among the incinerators. In PM_10_ PCA (Fig. 10a), Ag, Pt, Pd, and Au were largely loaded on the first principal component (PC1), which explained 60.89% of the total variance, while Rh separated to principal component 2 (PC2), which explained 21.4%. Since PM_10_ represents the fine particulate fraction entrained in the flue gas stream, it is likely that Ag, Au, Pd, and Pt originated from common emission sources within the incinerators. Pearson correlation analysis (Fig. 6a) indicated strong correlations for Ag, specifically with Pt (*r* = 0.81) and Pd (*r* = 0.87). Au demonstrated a moderate correlation with Ag (*r* = 0.56). In contrast, Rh showed a good correlation with Pt (*r* = 0.67) and Rh showed no meaningful correlation with the other metals (*r* ≈ 0). These data indicated a common source for Ag, Pt, Pd, and Au and that was municipal waste (IW2, IW4) mostly from small appliances, electronics, and cosmetics. The data showed that Rh was likely, predominantly from industrial waste (IW10, IW11).

For the fly ash (FA) samples (Fig. 10b), Ag, Au, and Pd loaded predominantly on PC1, which explained 51.15% of the variance, while Pt and Rh loaded on PC2, accounting for 29.17%. The relatively large vector magnitudes of Rh and Pt on both PC1 and PC2 indicate that their distributions are influenced by multiple contributing factors across IW samples, suggesting more complex partitioning behavior in fly ash. In contrast, the clustering of Au toward PC1 implies a relatively uniform distribution pattern, likely reflecting more consistent source characteristics among the investigated MSWI plants. Although PC1 and PC2 show characteristic loadings, PC1 dominated by Ag, Au and Pd, and PC2 more strongly influenced by Pt and Rh, the overlap of vector contributions, particularly for Pt and Rh, suggests that each component reflects multiple underlying factors rather than a single metal group. The PCA therefore indicates associative trends (*e.g.*, partial grouping of platinum-group elements) but does not support assigning formal descriptive names to PC1 or PC2. Pearson correlation analysis (Fig. 11a) further supported these groupings, showing a very strong correlation between Ag and Pd (*r* = 0.88), Pt and Rh(*r* = 0.94), Pd and Rh also had a moderate mutual correlation (Pd/Rh, *r* = 0.557). In contrast, Au showed no correlation with the remaining metals (*r* = 0.060–0.0227).

These findings indicated that Ag, Au, and Pd were susceptible to volatilization during waste incineration and subsequently condensed in the fly ash fraction. On the other hand, Pt and Rh – displaying higher thermal stability – appeared only partially in FA, and tended to be found in association with one another. The emission sources of these metals were predominantly associated with industrial waste (IW7, IW8).

In contrast to PM_10_ and FA, the results of the PCA analysis in BA (Fig. 10c) indicate that PC1 (39.45%) was dominated by Pt and Pd, while PC2 (33.75%) was associated with Rh and partially Au. Ag, on the other hand, is separate and not aligned with the Pt–Pd group. The correlation results (Fig. 11c) also show that Pt–Pd has a fairly tight relationship (*r* = 0.65), while Ag only has a very weak correlation with other metals (Ag/Pd, *r* = 0.19; Ag/Pt, *r* approx 0.02). This may be related to Pt and Pd having high thermal stability and low volatility, thus mainly accumulating in the bottom ash. Conversely, Ag is highly volatile, and most of it escapes with the flue gas or condenses in the fly ash, with only a small, dispersed amount remaining in the BA. Rh, originating from catalysts, appears sporadically and may be related to the flaking or deposition of catalytic material in the combustion chamber.

Thus, the PCA analysis combined with the correlation matrix has indicated the distribution trend of precious metals in the waste incinerator with different types of waste. The group of Ag, Au, Pd, Pt mainly originates from household electronics, alloys, chemicals, and cosmetics, while Rh reflects the influence of catalysts and chemicals. The difference in the volatility and thermal stability characteristics of each metal determines the distribution trend: Ag and Au concentrate in PM_10_ and FA, while Pt and Pd accumulate more in BA. However, in this study, some samples showed that Ag content in bottom ash was higher than in fly ash, especially in industrial waste. This could be related to the formation of refractory (low-volatility) Ag compounds (*e.g.*, AgCl, Ag_2_S, Ag alloys with Cu/Ni), which would not easily volatilize at the incinerator temperature.^50^ In that case, Ag is not dispersed with the flue gas but settles in the bottom ash, resulting in a higher Ag content in BA than FA.

### 3.5. Annual emission assessment of precious metals in incinerators

To evaluate the potential for precious metal recovery, the annual emissions (AE) of five target metals (Ag, Au, Pt, Pd, and Rh) were calculated for PM_10_, fly ash (FA), and bottom ash (BA) in the studied incinerators. The calculated annual emissions are summarized in Table 2.

**Table 2:** Assessment of the annual emission mass AE (g per year) of precious metals in BA, FA, and PM_10_, calculated based on the actual operating capacity of the studied waste incinerators

Incinerators	AE (g per year)						∑*H*_metals_ (Eur per year)
	Ag	Au	Pt	Pd	Rh	SUM	
**AE – PM**_**10**_ **(g per year)**							
IW1	8.66 × 10^1^	7.59 × 10^1^	—	1.60 × 10^1^	1.24 × 10^2^	3.03 × 10^2^	9.88 × 10^3^
IW2	8.80 × 10^2^	5.45 × 10^1^	8.65 × 10^2^	8.93 × 10^1^	9.39 × 10^1^	1.98 × 10^3^	1.65 × 10^4^
IW3	4.61 × 10^2^	6.22 × 10^2^	1.82 × 10^2^	6.60 × 10	2.04 × 10^2^	1.48 × 10^3^	2.96 × 10^4^
IW4	2.12 × 10^3^	4.91 × 10^3^	1.38 × 10^3^	3.20 × 10^1^	1.58 × 10^2^	8.59 × 10^3^	1.40 × 10^5^
IW5	4.35 × 10^3^	1.71 × 10^3^	1.40 × 10^3^	1.26 × 10^3^	2.49 × 10^2^	8.97 × 10^3^	8.26 × 10^4^
IW6	6.10 × 10^2^	1.09 × 10^2^	1.07 × 10^1^	2.47 × 10^1^	8.75 × 10^2^	1.63 × 10^3^	5.88 × 10^4^
IW7	3.67 × 10^2^	2.91 × 10^2^	4.60 × 10	1.09 × 10^2^	1.82 × 10^2^	9.54 × 10^2^	1.97 × 10^4^
IW8	8.09 × 10^2^	6.76 × 10^1^	1.51 × 10^2^	1.83 × 10^2^	1.56 × 10^2^	1.37 × 10^3^	1.50 × 10^4^
IW9	1.21 × 10^3^	9.92 × 10^1^	—	2.95 × 10^1^	1.83 × 10^2^	1.52 × 10^3^	1.49 × 10^4^
IW10	1.65 × 10^3^	8.61 × 10^2^	1.05 × 10^1^	3.50 × 10	5.04 × 10^2^	3.03 × 10^3^	5.34 × 10^4^
IW11	1.21 × 10^2^	1.01 × 10^2^	5.90 × 10	2.95 × 10^2^	1.95 × 10^2^	7.19 × 10^2^	1.76 × 10^4^
Mean	1.15 × 10^3^	8.09 × 10^2^	3.65 × 10^2^	1.86 × 10^2^	2.66 × 10^2^	2.78 × 10^3^	4.16 × 10^4^

**AE – FA (g per year)**							
IW1	1.29 × 10^1^	2.77 × 10^−1^	1.70 × 10^−1^	4.26 × 10^−1^	3.70 × 10^−3^	1.37 × 10^1^	1.94 × 10^1^
IW2	6.01 × 10^−1^	2.70 × 10^−2^	—	1.38 × 10^−1^	1.00 × 10^−4^	7.66 × 10^−1^	2.19 × 10
IW3	6.20 × 10^−1^	6.00 × 10^−3^	—	1.90 × 10^−2^	2.00 × 10^−4^	6.46 × 10^−1^	6.75 × 10^−1^
IW4	1.15 × 10^1^	8.13 × 10	9.00 × 10^−2^	4.50 × 10^−2^	6.60 × 10^−3^	1.97 × 10^1^	2.00 × 10^2^
IW5	1.71 × 10^1^	3.33 × 10^−1^	2.50 × 10^−1^	2.64 × 10^−1^	3.20 × 10^−3^	1.79 × 10^1^	2.23 × 10^1^
IW6	1.08 × 10^1^	2.00 × 10	2.72 × 10^−1^	1.36 × 10^−1^	2.70 × 10^−3^	1.32 × 10^1^	5.70 × 10^1^
IW7	2.90 × 10	0.00 × 10	4.36 × 10	2.32 × 10	1.37 × 10^−2^	9.59 × 10	6.30 × 10^1^
IW8	1.47 × 10^1^	8.50 × 10^−2^	1.21 × 10^−1^	1.25 × 10	1.00 × 10^−4^	1.61 × 10^1^	2.25 × 10^1^
IW9	1.95 × 10	4.10 × 10^−2^	2.90 × 10^−2^	1.70 × 10^−2^	1.00 × 10^−4^	2.04 × 10	2.48 × 10
IW10	6.50 × 10^−2^	6.00 × 10^−2^	—	1.90 × 10^−2^	1.00 × 10^−4^	1.44 × 10^−1^	1.62 × 10
Mean	7.31 × 10	1.10 × 10	5.29 × 10^−1^	4.63 × 10^−1^	3.00 × 10^−3^	9.40 × 10	3.91 × 10^1^

**AE – BA (g peryear)**							
IW1	2.63 × 10^3^	1.65 × 10^1^	2.48 × 10^1^	1.07 × 10^2^	0.00 × 10	2.78 × 10^3^	3.07 × 10^3^
IW2	6.65 × 10^2^	2.79 × 10^1^	1.12 × 10	5.77 × 10^1^	0.00 × 10	7.52 × 10^2^	1.56 × 10^3^
IW3	7.65 × 10^2^	1.10 × 10^1^	1.83 × 10^2^	4.39 × 10^2^	0.00 × 10	1.40 × 10^3^	6.27 × 10^3^
IW4	4.05 × 10^3^	1.03 × 10^4^	—	5.83 × 10^3^	6.22 × 10^1^	2.02 × 10^4^	3.00 × 10^5^
IW5	5.12 × 10^4^	1.94 × 10^2^	—	1.78 × 10^3^	9.83 × 10	5.32 × 10^4^	4.98 × 10^4^
IW6	1.99 × 10^4^	1.18 × 10^3^	7.45 × 10^2^	1.08 × 10^3^	2.85 × 10	2.29 × 10^4^	5.57 × 10^4^
IW7	3.90 × 10^4^	3.14 × 10^2^	2.83 × 10^3^	7.18 × 10^2^	4.70 × 10^1^	4.29 × 10^4^	6.46 × 10^4^
IW8	6.34 × 10^3^	8.84 × 10^1^	1.96 × 10^2^	1.96 × 10^2^	0.00 × 10	6.82 × 10^3^	9.20 × 10^3^
IW9	7.36 × 10^3^	2.62 × 10^1^	2.33 × 10^1^	1.40 × 10^2^	0.00 × 10	7.54 × 10^3^	6.21 × 10^3^
IW10	1.81 × 10^2^	1.36 × 10^2^	4.52 × 10^1^	5.42 × 10^1^	0.00 × 10	4.16 × 10^2^	4.20 × 10^3^
IW11	7.15 × 10^4^	2.72 × 10^3^	1.36 × 10^3^	7.77 × 10^2^	—	7.63 × 10^4^	1.24 × 10^5^
Mean	1.85 × 10^4^	1.36 × 10^3^	4.91 × 10^2^	1.02 × 10^3^	1.12 × 10^1^	2.14 × 10^4^	5.68 × 10^4^

The mean AE values followed the order FA < PM10 < BA, ranging from 1.86 × 10^2^ to 1.15 × 10^3^ g per year in PM10, 0.003 to 7.305 g per year in FA, and 1.12 × 10^1^ to 1.36 × 10^3^ g per year in BA. These results indicated that the majority of precious metal emissions accumulated in BA, primarily due to the much larger mass of bottom ash, which is approximately 900–1000 times higher than fly ash emissions.^36^ For example, 1 ton of waste generated roughly 230 kg of bottom ash.^51^

**Fig. 12:**
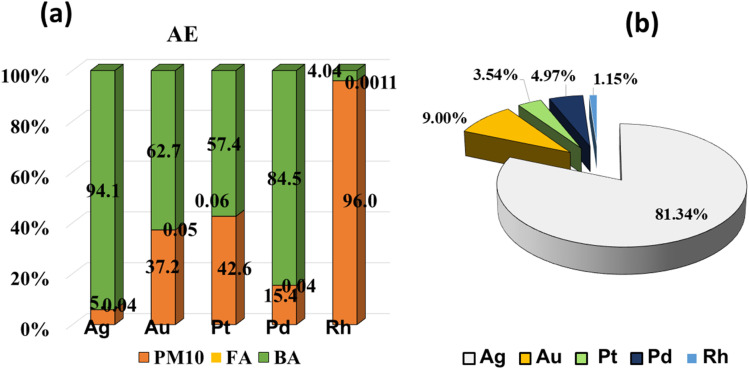
Average annual emissions of precious metals in PM10, FA, and BA from the waste incinerator (a); total emissions of precious metals from the incinerator (b).

The percentage distribution of AE further confirmed that nearly all metals were concentrated ranging from 4.04% to 94.1% in BA, 5.86–96% in PM10, whereas their contributions in FA and were very low, approximately 0.001–0.06% (Fig. 12a). The AE of PM10 accounts for a fairly high proportion of the exhaust gas flow rate (13 000–20 000 Nm^3^ h^−1^). PM_10_ exists in the entire volume of this gas, while FA is only the part retained by the filter bag (Table S1). The total mean annual emissions of the metals followed the sequence: Ag (81,34%) > Au (9%) > Pd (4.97%) > Pt (3.54%) > Rh (1.15%) (Fig. 12b).

The total AE values of precious metals in this study were lower than those reported by Morf *et al.* (2013) at the Kezo incinerator (Au: 80 ± 40 kg per year) and Swiss municipal incinerators (Au: 1.3 t per year).^8^ Nevertheless, the annual emission of each metal strongly depended on incinerator capacity and waste throughput.

### 3.6. Predicted economic potential from precious metal recovery in incinerators

This study proposed two approaches to estimate the economic potential of recovering precious metals from incinerator residues (Tables 2 and 3). The results of the first approach were presented in Table 2, based on the total *H*_metals_ value for each incinerator.

**Table 3:** Assessment of the recovery potential of precious metals in BA, FA, and PM10 from waste incinerators

Incinerators	*Q*_metal_ (Eur per year)						ANCF (Eur per year)
	Ag	Au	Pt	Pd	Rh	SUM	
**PM**_**10**_							
IW1	3.26 × 10^2^	1.20 × 10^4^	—	6.95 × 10^2^	5.29 × 10^4^	6.59 × 10^4^	−3.51 × 10^5^
IW2	3.27 × 10^3^	8.48 × 10^3^	5.23 × 10^4^	3.82 × 10^3^	3.94 × 10^4^	1.07 × 10^5^	−3.10 × 10^5^
IW3	1.71 × 10^3^	9.68 × 10^4^	1.10 × 10^4^	2.83 × 10^2^	8.57 × 10^4^	1.96 × 10^5^	−2.21 × 10^5^
IW4	5.18 × 10^3^	5.03 × 10^5^	5.49 × 10^4^	9.02 × 10^2^	4.36 × 10^4^	6.07 × 10^5^	1.91 × 10^5^
IW5	1.86 × 10^4^	3.06 × 10^5^	9.77 × 10^4^	6.18 × 10^4^	1.20 × 10^5^	6.04 × 10^5^	1.87 × 10^5^
IW6	2.35 × 10^3^	1.75 × 10^4^	6.70 × 10^2^	1.09 × 10^3^	3.80 × 10^5^	4.02 × 10^5^	−1.51 × 10^4^
IW7	1.41 × 10^3^	4.69 × 10^4^	2.87 × 10^2^	4.83 × 10^3^	7.93 × 10^4^	1.33 × 10^5^	−2.84 × 10^5^
IW8	3.05 × 10^3^	1.07 × 10^4^	9.26 × 10^3^	7.92 × 10^3^	6.63 × 10^4^	9.72 × 10^4^	−3.20 × 10^5^
IW9	4.56 × 10^3^	1.56 × 10^4^	—	1.28 × 10^3^	7.77 × 10^4^	9.92 × 10^4^	−3.18 × 10^5^
IW10	5.76 × 10^3^	1.26 × 10^5^	5.97 × 10^2^	1.39 × 10^2^	1.99 × 10^5^	3.32 × 10^5^	−8.51 × 10^4^
IW11	4.67 × 10^2^	1.63 × 10^4^	3.71 × 10^2^	1.31 × 10^4^	8.49 × 10^4^	1.15 × 10^5^	−3.02 × 10^5^
Mean	4.24 × 10^3^	1.05 × 10^5^	2.06 × 10^4^	8.71 × 10^3^	1.12 × 10^5^	2.51 × 10^5^	−1.66 × 10^5^

**FA**							
IW1	1.13 × 10^5^	1.02 × 10^5^	2.44 × 10^4^	5.91 × 10^4^	8.33 × 10^4^	3.82 × 10^5^	−3.54 × 10^4^
IW2	1.05 × 10^5^	1.95 × 10^5^	—	3.81 × 10^5^	7.54 × 10^4^	7.57 × 10^5^	3.40 × 10^5^
IW3	1.09 × 10^5^	4.51 × 10^4^	—	5.29 × 10^4^	1.62 × 10^5^	3.69 × 10^5^	−4.80 × 10^4^
IW4	6.77 × 10^4^	2.01 × 10^6^	8.68 × 10^3^	4.21 × 10^3^	1.51 × 10^5^	2.24 × 10^6^	1.82 × 10^6^
IW5	2.41 × 10^5^	1.96 × 10^5^	5.73 × 10^4^	5.86 × 10^4^	6.63 × 10^4^	6.19 × 10^5^	2.02 × 10^5^
IW6	2.08 × 10^5^	1.61 × 10^6^	8.55 × 10^4^	4.14 × 10^4^	1.19 × 10^5^	2.06 × 10^6^	1.65 × 10^6^
IW7	5.58 × 10^4^	—	1.37 × 10^6^	7.05 × 10^5^	5.98 × 10^5^	2.73 × 10^6^	2.31 × 10^6^
IW8	1.32 × 10^6^	3.18 × 10^5^	1.77 × 10^5^	1.77 × 10^6^	2.99 × 10^5^	3.88 × 10^6^	3.47 × 10^6^
IW9	1.75 × 10^5^	1.55 × 10^5^	4.22 × 10^4^	2.34 × 10^4^	1.26 × 10^5^	5.22 × 10^5^	1.05 × 10^5^
IW10	4.57 × 10^3^	1.76 × 10^5^	—	2.08 × 10^4^	8.14 × 10^4^	2.82 × 10^5^	−1.35 × 10^5^
Mean	2.40 × 10^5^	4.80 × 10^5^	1.76 × 10^5^	3.12 × 10^5^	1.76 × 10^5^	1.38 × 10^6^	9.67 × 10^5^

**BA**							
IW1	2.42 × 10^5^	6.36 × 10^4^	3.71 × 10^4^	1.56 × 10^5^	8.58 × 10^4^	5.84 × 10^5^	1.67 × 10^5^
IW2	1.23 × 10^5^	2.17 × 10^5^	3.37 × 10^3^	1.69 × 10^5^	1.40 × 10^5^	6.52 × 10^5^	2.35 × 10^5^
IW3	1.42 × 10^5^	8.50 × 10^4^	5.51 × 10^5^	1.28 × 10^6^	1.91 × 10^5^	2.25 × 10^6^	1.83 × 10^6^
IW4	2.06 × 10^4^	2.19 × 10^6^	—	4.68 × 10^5^	1.22 × 10^6^	3.90 × 10^6^	3.48 × 10^6^
IW5	5.65 × 10^5^	8.97 × 10^4^	—	3.10 × 10^5^	1.61 × 10^5^	1.13 × 10^6^	7.09 × 10^5^
IW6	1.45 × 10^5^	3.63 × 10^5^	8.89 × 10^4^	1.25 × 10^5^	4.72 × 10^4^	7.70 × 10^5^	3.53 × 10^5^
IW7	2.86 × 10^5^	9.63 × 10^4^	3.37 × 10^5^	8.30 × 10^4^	7.80 × 10^5^	1.58 × 10^6^	1.17 × 10^6^
IW8	5.83 × 10^5^	3.40 × 10^5^	2.94 × 10^5^	2.85 × 10^5^	3.99 × 10^5^	1.90 × 10^6^	1.48 × 10^6^
IW9	6.76 × 10^5^	1.01 × 10^5^	3.48 × 10^4^	2.03 × 10^5^	6.05 × 10^4^	1.08 × 10^6^	6.58 × 10^5^
IW10	1.41 × 10^4^	4.42 × 10^5^	5.73 × 10^4^	6.66 × 10^4^	1.59 × 10^5^	7.39 × 10^5^	3.22 × 10^5^
IW11	5.24 × 10^5^	8.35 × 10^5^	1.62 × 10^5^	8.99 × 10^4^	1.61 × 10^5^	1.77 × 10^6^	1.35 × 10^6^
Mean	3.02 × 10^5^	4.39 × 10^5^	1.42 × 10^5^	2.94 × 10^5^	3.10 × 10^5^	1.49 × 10^6^	1.07 × 10^6^

**Fig. 13:**
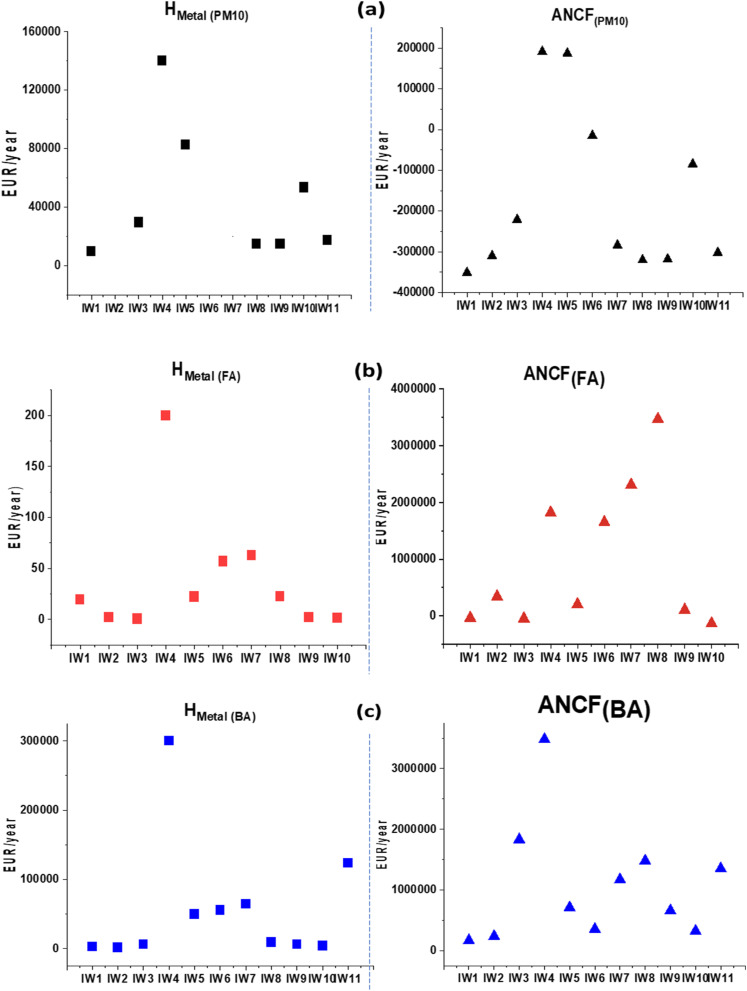
Predicted economic potential of recovering precious metals from PM10 (a), fly ash (FA, (b)), and bottom ash (BA, (c)) in municipal solid waste incinerators.

Scenario 1 considered only the theoretical economic benefit, calculated from the market prices of the precious metals at the time of the study. The total *H*_metals_ value for each incinerator strongly depended on its operational capacity (see Table S1 in SI). On average, the total *H*_metals_ value was highest in bottom ash, reaching 5.68 × 10^4^ EUR per year, which was 1.364 EUR higher than in PM_10_ (4.16 × 10^4^ EUR per year) and 1454 EUR higher than in fly ash (3.91 × 10^1^ EUR per year) (Table 2 and Fig. 13).

The results in Table 2 showed the average value of the *H*_metals_ in PM_10_ were 4.16 × 10^4^ EUR per year (range: 9.88 × 10^3^–1.40 × 10^5^ EUR per year). This means that the economic potential and resilience are quite high. The estimated value of fly ash ranged from 6.75 × 10^−1^ to 2.00 × 10^2^ per year. The value depended on the operating capacity, average emission flow rate and the amount of ash from each incinerator. For example, IW4 produced 2.00 × 10^2^ EUR per year, and IW7 produced 6.30 × 10^1^ EUR per year. Fly ash recovery potential exists, despite levels being lower than PM10. Investment in enhanced treatment capacity and recovery efficiency could increase its value.

For bottom ash, the estimated economic values were similar among the incinerators. Some industrial waste plants reached values of 3.00 × 10^5^ EUR per year for IW4 and 1.24 × 10^5^ EUR per year for IW11. These results show that recovering precious metals from bottom ash has good economic potential. This applies to both municipal and industrial waste incinerators.

In Scenario 2, the economic worth of recovering precious metals was estimated and corrected for investment deprecation and operational expenses, based on the Hinwil model plant in Switzerland,^9^ excluding payback time as well as profit margins. As demonstrated in Table 3, calculation showed that after deducting these expenses, the economic worth for PM10 was virtually impossible to achieve, with 82% of the incinerators becoming negative values (average: −1.66 × 10^5^ EUR per year). The estimated mean ANCF for fly ash was 9.67 × 10^5^ EUR per year, 70% of the instances providing positive values between 1.05 × 10^5^ and 3.47 × 10^6^ EUR per year. For bottom ash, all incinerators provided positive ANCF values with a mean of 1.07 × 10^6^ EUR per year (range: 1.67 × 10^5^–3.48 × 10^6^ EUR per year). The findings demonstrated that both the economic rewards estimated for fly ash and bottom ash were feasible.

The incinerators in Vietnam are operating at relatively low capacities (6570–8030 h per year) with smaller and lower-technology scales, and therefore the recovery efficiencies are low. The two scenarios explicitly indicated that scaling up plant capacity to >50 000 t per year and adopting advanced, large-scale processing technologies (only 28.8–057.4% in the model's recovery efficiency) would render both bottom ash and fly ash economically feasible and investment-worthy.^51^Fig. 13 also reveals a clear trend under Scenario 1 (*H*_metal_), in which the recovery potential is strongly dependent on the actual operating capacities of individual incinerators. The municipal solid waste incinerator IW4 and the mixed-waste incinerators IW5 and IW6 exhibit the highest and most consistent recovery potential across PM_10_, FA, and BA. Among the industrial incinerators, IW7 and IW8 demonstrate strong recovery potential for both FA and BA, whereas IW10 and IW11 are more favorable for PM_10_ recovery. These differences are largely attributable to variations in incineration operating conditions. In contrast, under Scenario 2 (ANCF), when a unified techno-economic framework based on the Hinwil plant is applied, a relatively homogeneous recovery potential is observed across all three matrices (PM_10_, FA, and BA). The most promising facilities in this scenario include the municipal incinerator IW4, the mixed-waste incinerator IW5, and the industrial incinerators IW7 through IW11.

Previous studies supported these data. Muchova *et al.* indicated net incomes of 292 and 15 860 EUR per kg for Ag and Au recovery from bottom ash after deducting investment and processing costs.^13^ These results identified that long-term investment in precious metal recovery projects must balance economic return with investment cost and environmental sustainability.

It should be noted that Scenario 1 represents a conservative and more realistic estimate because it is based on actual recovery efficiencies currently achieved at MSWI facilities (with conservative assumptions for Pt and Pd). By contrast, Scenario 2 assumes 100% metal recovery and therefore represents a theoretical upper-bound potential rather than an operationally attainable value. For practical decision-making, Scenario 1 provides the more reliable basis, whereas Scenario 2 highlights the long-term strategic potential if advanced recovery technologies are fully implemented in the future.

### 3.7. Implications for environmental management

The investigation identified key problems were associated with environmental management and sustainable waste management. High levels of Ag, Au, Pt, Pd, and Rh were identified in bottom ash, and lower levels in fly ash and PM_10_ indicated that incineration residue might not have been disposed of properly and metals released could potentially leach to soil, water bodies, and contaminate the atmosphere. While generally less toxic than other base metals such as Pb or Cd, the persistence and potential bioaccumulation of noble metals could have posed an environmental risk because of its locational proximity to the disposal sites. Fine particulate matter (PM_10_) containing volatilizable metals such as Ag and Au may have shared to atmospheric dispersion when species were released and inhalable risk to workers or residents living in neighbourhood. The study also indicated the importance of monitoring the combustion of flue gas and the residual solid waste.

The bottom ash served as a concentrated source of recoverable metals, with silver (Ag) accounting for over 90% of the total annual releases. This finding suggests that recovery processes could both reduce environmental pollution and generate economic value. Recovery techniques such as hydrometallurgical leaching and mechanical sorting were considered practical, depending on the characteristics of the residue. Fly ash, being dense and glass-like, required chemical pre-treatment, while bottom ash, which was coarse and irregular, allowed for simpler recovery methods.

Overall, the study emphasized the importance of aligning policies on waste incineration management with resource recovery strategies. Establishing clear regulations for the safe handling, storage, and treatment of residues, together with investment in advanced recovery technologies and efficient incineration systems, could enhance both environmental protection and economic sustainability. Such measures would ensure safer resource utilization while reducing environmental risks when consistent with circular economy principles and responsible resource management.

### 3.8. Limitation and future work

Although the present work focuses on quantifying metal distribution and theoretical recovery potential, MSWI residues can be processed using established metals-extraction pathways. A feasible strategy typically begins with physical pretreatment (drying, sieving, magnetic separation, and density classification) to isolate metal-rich fractions and reduce reagent consumption. Hydrometallurgical leaching routes (*e.g.*, aqua regia, thiourea, or chloride media) or selective precipitation can then be applied to recover Ag, Au, Pt, Pd, and Rh, with optimization commonly involving adjustments in particle size, pH, oxidant concentration, temperature, and contact time. However, hydrometallurgical and pyrometallurgical processes were not included in the present economic assessment because no MSWI facility in Vietnam currently operates these technologies, and validated operational data (recovery yields, reagent requirements, energy use, and waste-treatment costs) are therefore unavailable. Using generic parameters from foreign facilities would introduce substantial uncertainty, as process performance and economics vary strongly with scale and residue characteristics. Future work should incorporate process-specific economic modelling and optimized extraction parameters once reliable Vietnam-specific operational data become available.

## 4. Conclusions

This study provided one of the most intensive investigations of the concentration, distribution, and recovery potential of five precious metals (Ag, Au, Pt, Pd, and Rh) in all waste streams (fine particulate matter (PM10), fly ash (FA), and bottom ash (BA)) of municipal solid waste incinerators (MSWI) in Northern Vietnam. The study included three waste types: household, mixed (30% household + 70% industrial), and industrial to distinguish possible sources of emissions for each metal and recovery options. All target metals detected in PM10, FA, and BA, and concentrations generally followed the order of BA > FA > PM10. Silver (Ag) was the most abundant metal reported and was found >99% of the time in BA, followed by Au, Pd, and Pt, with Rh occurring at very low levels. Au was detected greater in the municipal and mixed waste, whereas Pd, Pt, and Rh were primarily from industrial waste. Significant correlations among metals (*e.g.*, Ag/Pd *r* = 0.87, Au/Pt *r* = 0.67, Ag/Pt *r* = 0.81, Pt/Pd *r* > 0.6, Pt/Rh *r* = 0.94) suggested potential co-occurrence and common emission sources within specific waste fractions.

Economic evaluation under two different scenarios showed that both fly ash and bottom ash have strong potential for metal recovery. In the first scenario, which assumed ideal recovery efficiency, the results suggested notable economic gains. In the second scenario, where investment and operating expenses were considered, the analysis still indicated reasonable net profits, supporting long-term feasibility. In general, the results confirm that recovering precious metals from incineration residues, especially from bottom ash, can be economically practical.

On the whole, the work provides a reference data set for Vietnamese incinerator ashes for precious metal contents, sheds light on the possibility of valorization of wastes and secondary sources of major metals, and supports circular economy and safe waste disposal strategies. Metal speciation must be a subject of future studies and designing of effective hydrometallurgical or pyrometallurgical technology for preferential metal recovery so that recovery effectiveness and environmental sustainability are enhanced further.

## Author contributions

Thi Thu Thuy Nguyen engaged in the conceptualization, methodology, and manuscript preparation. Truong Xuan Vuong contributed significantly to data interpretation and writing and editing manuscript. Thi Thu Ha Pham, Anh Quoc Hoang, Minh Binh Tu, and Thi Hue Nguyen contributed equally to data collection, analysis, and manuscript review. All authors approved the final version of the manuscript.

## Conflicts of interest

The authors declare no conflicts of interest.

## Supplementary Material

RA-016-D5RA08421K-s001

## Data Availability

The data supporting this article have been included as part of the supplementary information (SI). Supplementary information: additional data supporting this study, including: (i) detailed information on municipal and industrial waste incinerators in northern Vietnam, such as location, operating conditions, waste types, and ash generation rates (Table S1); (ii) quality control and analytical validation results for Ag, Au, Pt, Pd, and Rh determination, including recovery efficiencies and repeatability evaluated using certified reference materials (Table S2); (iii) percentage distribution of target precious metals among PM_10_, fly ash, and bottom ash collected from different incinerators (Table S3); and (iv) one-way ANOVA and Fisher's *post hoc* test results assessing statistically significant differences in precious metal contents across sample matrices (PM_10_, fly ash, and bottom ash) (Table S4). See DOI: https://doi.org/10.1039/d5ra08421k.
