# Supplementary material for: Comprehensive assessment of precious metal concentration, distribution, and recovery potential in municipal solid waste incineration residues from northern Vietnam
Source: RSC Adv. 2026 Jan 13;16(4):2937–57. doi: 10.1039/d5ra08421k (PMC12797203; doi:10.1039/d5ra08421k)
Supplement: RA-016-D5RA08421K-s001 [file RA-016-D5RA08421K-s001.pdf]

Supplementary Information (SI) for RSC Advances.
This journal is © The Royal Society of Chemistry 2026

SUPPLEMENTARY INFORMATION

Table S1. Information on waste incinerators collected in some provinces in the North of Vietnam

No	Sample site	incinerators	Location	P (ton/h) Incineration Capacity	AOT (h/year) Average operating time	GR_{FA} (kg/ton) Fly Ash generation rate	GR_{BA} (kg/ton) Bottom Ash generation rate	F Average emission flow rate (Nm^3/h)	Type of waste
Municipal waste									
1	Bac Ninh	IW1	21.15395700160616,106.23798604547376	3.0	8030	0.133	12.7	13000	100% municipal waste (plastic, packaging, food waste, home electronics, jewelry, Scrap metal, personal waste,)
2	Hai Phong	IW2	20.90114422060879, 106.61355932284717	1.0	7300	0.022	20.8	14500	
3		IW3	20.90114422060879, 106.61355932284717	1.0	7300	0.022	20.8	14500	
4	Ha Noi	IW4	21.001887081469857, 105.79455775231595	4.5	8030	0.132	153	20000	
Industrial waste (70%) + Municipal waste (30%)									
5	Phu Tho	IW5	20.50352913924398, 105.79950579144753	2.0	6570	0.152	194	14000	30% municipal waste + 70% industrial
6	Bac Ninh	IW6	21.019493883744186, 106.05243119620057	2.0	7300	0.100	263	14000	
Industrial waste									
7	Bac Ninh	IW7	21.067434671320942,	2.0	7300	0.100	263	14000	100%

No	Sample site	incinerators	Location	P (ton/h) Incineration Capacity	AOT (h/year) Average operating time	GR_{FA} (kg/ton) Fly Ash generation rate	GR_{BA} (kg/ton) Bottom Ash generation rate	F Average emission flow rate (Nm^3/h)	Type of waste
Municipal waste									
			106.25434724964013						industrial waste (such as nylon, cloth, paper, pressed mud, plastic, Scrap metal, rags, iron filings, dry grease. chemical)
8		IW8	21.029169751945986, 105.82522425634284	3.0	8030	0.013	12.7	13000	
9		IW9	21.15395700160616,106.23798604547376	3.0	8030	0.013	12.7	13000	
10		IW11	21.15309653373626, 105.89937055520124	2.0	7300	-	263	14000	
11	Hai Phong	IW10	20.77970876572024, 106.38404262574647	2.5	8030	0.02	18	14000	

Table S2. Results of the recovery and repeatability evaluation of Ag, Au, Pt, Pd, and Rh compared with the certified reference material

**Precious metals**	**concentration (µg/kg)**	**Mean (µg/kg)**	**RSD%**	**Certificate Value BCR 723 (µg/kg)**	**Certificate Value 2709 (µg/kg)**	**Average recovery efficiency (%)**	**Reference**
^{109}Ag	348 - 406	372	8.1	-	410 ± 30	90.7	[1]
^{197}Au	277 - 298	284	4.17	-	300	94.7	[1]
^{195}Pt	80.7 - 86.4	85.1	4.65	81.3±2.5	-	105	[2,3]
^{105}Pd	6.22 - 6.82	6.49	4.69	6.1±1.9	-	106	[2,3]
^{103}Rh	11.9 - 14.2	13.1	8.6	12.8 ±1.3	-	103	[2,3]

Reference

1. Gills, T. E., & Kane, J. S. (1993). Certificate of analysis, standard reference material 2711. *National Institute of Standards and Technology, Gaithersburg, MD*. [Online]. Available. https://tsapps.nist.gov/srmext/certificates/archives/2709.pdf
2. Zischka, M., Schramel, P., Muntau, H., Rehnert, A., Gomez, M. G., Stojanik, B., ... & Maier, E. A. (2002). A new certified reference material for the quality control of palladium, platinum and rhodium in road dust, BCR-723. *TrAC Trends in Analytical Chemistry*, *21*(12), 851-868.
3. Hsu, W. H., Jiang, S. J., & Sahayam, A. C. (2013). Determination of Pd, Rh, Pt, Au in road dust by electrothermal vaporization inductively coupled plasma mass spectrometry with slurry sampling. *Analytica Chimica Acta*, *794*, 15-19.

Table S3: Percentage distribution of target metals (Ag, Au, Pt, Pd, and Rh) in PM10, FA, BA

	Ratio %				
**Incinerators**	**Ag**	**Au**	**Pt**	**Pd**	**Rh**
PM10					
IW1	29	25	nd	5	41
IW2	44	3	44	5	5
IW3	31	42	12	0.4	14
IW4	25	57	16	0.4	2
IW5	49	19	16	14	3
IW6	37	7	1	2	54
IW7	38	31	0.5	11	19
IW8	59	5	11	13	11
IW9	80	7	nd	2	12
IW10	54	28	0.3	0.1	17
IW11	17	14	0.8	41	27
FA					
IW1	93	2.0	1.2	3.1	0.6
IW2	78	3.5	nd	18	0.5
IW3	95	0.9	nd	2.9	1.3
IW4	57	41	0.5	0.2	1.1
IW5	95	1.9	1.4	1.5	0.2
IW6	81	15	2.1	1.0	0.4
IW7	29	nd	44	24	2.8
IW8	91	0.5	0.8	7.7	0.2
IW9	95	2.0	1.4	0.8	0.6
IW10	42	39	nd	12	6.7
BA					
IW1	94	0.6	0.9	3.9	0.3

IW2	88	3.7	0.1	7.6	0.9
IW3	54	0.8	13	31	0.6
IW4	18	46	nd	26	9.5
IW5	96	0.4	nd	3.3	0.2
IW6	87	5.2	3.3	4.7	0.2
IW7	89	0.7	6.5	1.6	2.2
IW8	92	1.3	2.9	2.9	0.6
IW9	97	0.3	0.3	1.9	0.1
IW10	42	31	10	13	4.2
IW11	93	3.6	1.8	1.0	0.3

nd: "not detection"

Table S4. One-way ANOVA results of the precious metal content in PM10 (a), FA (b), and BA (c)

(a) Fisher Test PM10

	Index	Mean Difference	Std. Error	DF	|t|value	Prob>|t|	Alpha	Sig Flag	95.00% LCL	95.00% UCL
Ag Au	0	4.39439	2.6645	40	1.64924	0.10693	0.05	0	-0.99076	9.77955
Ag Pt	1	7.57201	2.6645	40	2.84181	0.00703	0.05	1	2.18685	12.95717
Ag Pd	2	8.87377	2.6645	40	3.33037	0.00187	0.05	1	3.48862	14.25893
Ag Rh	3	8.28186	2.6645	40	3.10822	0.00346	0.05	1	2.89671	13.66702
Au Pt	4	3.17762	2.6645	40	1.19258	0.24006	0.05	0	-2.20754	8.56277
Au Pd	5	4.47938	2.6645	40	1.68113	0.10053	0.05	0	-0.90578	9.86453
Au Rh	6	3.88747	2.6645	40	1.45899	0.15238	0.05	0	-1.49768	9.27263
Pt Pd	7	1.30176	2.6645	40	0.48856	0.62782	0.05	0	-4.0834	6.68692
Pt Rh	8	0.70985	2.6645	40	0.26641	0.79129	0.05	0	-4.6753	6.09501
Pd Rh	9	-0.59191	2.6645	40	0.22215	0.82533	0.05	0	-5.97706	4.79325

(b) Fisher Test FA

	Index	Mean Difference	Std. Error	DF	|t|value	Prob>|t|	Alpha	Sig Flag	95.00% LCL	95.00% UCL
Ag Au	0	8.11438	2.7082	36	2.99623	0.00493	0.05	1	2.6219	13.60685
Ag Pt	1	8.1372	2.7082	36	3.00466	0.00482	0.05	1	2.64473	13.62968
Ag Pd	2	7.8201	2.7082	36	2.88757	0.00653	0.05	1	2.32763	13.31258
Ag Rh	3	8.46685	2.7082	36	3.12638	0.00349	0.05	1	2.97437	13.95933
Au Pt	4	0.02283	2.7082	36	0.00843	0.99332	0.05	0	-5.46965	5.5153
Au Pd	5	-0.29427	2.7082	36	0.10866	0.91408	0.05	0	-5.78675	5.1982
Au Rh	6	0.35248	2.7082	36	0.13015	0.89717	0.05	0	-5.14	5.84495
Pt Pd	7	-0.3171	2.7082	36	0.11709	0.90744	0.05	0	-5.80958	5.17538
Pt Rh	8	0.32965	2.7082	36	0.12172	0.9038	0.05	0	-5.16283	5.82213
Pd Rh	9	0.64675	2.7082	36	0.23881	0.81261	0.05	0	-4.84573	6.13922

(c) Fisher Test FA

	Index	Mean Difference	Std. Error	DF	|t|value	Prob>|t|	Alpha	Sig Flag	95.00% LCL	95.00% UCL
Ag Au	0	10.3569	1.66763	40	6.21056	2.39052E-7	0.05	1	6.9865	13.7273
Ag Pt	1	10.41856	1.66763	40	6.24753	2.12093E-7	0.05	1	7.04816	13.78896
Ag Pd	2	10.06643	1.66763	40	6.03637	4.20136E-7	0.05	1	6.69602	13.43683
Ag Rh	3	10.63176	1.66763	40	6.37537	1.40257E-7	0.05	1	7.26135	14.00216
Au Pt	4	0.06166	1.66763	40	0.03697	0.97069	0.05	0	-3.30874	3.43206
Au Pd	5	-0.29047	1.66763	40	0.17418	0.8626	0.05	0	-3.66088	3.07993
Au Rh	6	0.27486	1.66763	40	0.16482	0.86992	0.05	0	-3.09555	3.64526
Pt Pd	7	-0.35213	1.66763	40	0.21116	0.83384	0.05	0	-3.72253	3.01827
Pt Rh	8	0.2132	1.66763	40	0.12784	0.89891	0.05	0	-3.15721	3.5836
Pd Rh	9	0.56533	1.66763	40	0.339	0.73638	0.05	0	-2.80507	3.93573
